# Estimating SARS-CoV-2 exposure in asymptomatic hospitalized children with cancer in Western Kenya: A retrospective analysis of serological data

**DOI:** 10.1371/journal.pone.0353284

**Published:** 2026-07-10

**Authors:** Katarina M. DiLillo, Catherine S. Forconi, Angela Matta, Jeni Melo, Ryan P. McNamara, Ronald Tonui, Festus N’juguna, Juliana A. Otieno, Boaz Odwar, Ann Kinyua, Douglas A. Lauffenburger, Ann M. Moormann

**Affiliations:** 1 Department of Biological Engineering, Massachusetts Institute of Technology, Cambridge, Massachusetts, United States of America; 2 Division of Infectious Diseases and Immunology, Department of Medicine, University of Massachusetts Chan Medical School, Worcester, Massachusetts, United States of America; 3 Department of Immunology and Infectious Diseases, Harvard T.H. Chan School of Public Health, Boston, Massachusetts, United States of America; 4 Department of Pathology, Moi University, Eldoret, Kenya; 5 Department of Child Health and Pediatrics, Moi University, Eldoret, Kenya; 6 Ministry of Health, Jaramogi Oginga Odinga Teaching and Referral Hospital, Kisumu, Kenya; 7 Center for Global Health Research, Kenya Medical Research Institute, Kisumu, Kenya; University of Virginia, UNITED STATES OF AMERICA

## Abstract

**Background:**

During the COVID-19 pandemic, concerns emerged that hospital-based care could increase the risk of SARS-CoV-2 infection among pediatric patients with cancer, especially in resource-limited settings where the capacity to isolate patients would be challenging. Yet, infection rates in this population remain poorly documented, and it is unclear whether prior common human coronavirus (HCoV) exposures influence SARS-CoV-2 antibody responses. Here, we used serology to estimate SARS-CoV-2 exposure in children with and without cancer from Western Kenya and evaluated responses to common HCoVs to explore cross-reactivity.

**Methods:**

We performed multiplex antibody profiling to assess SARS-CoV-2 and HCoV-specific responses in plasma from children with and without cancer sampled between 2014 and 2022. Unsupervised clustering identified participants with elevated SARS-CoV-2 seroreactivity relative to pre-pandemic samples. Seropositivity was further defined using a multi-antigen approach based on IgG levels to nucleocapsid and receptor-binding domain antigens to improve specificity. Cross-reactivity and cross-boosting by common HCoVs were evaluated through correlation analyses and groupwise comparisons of antibody levels, respectively.

**Results:**

Of 564 children, 474 were healthy (184 pre-pandemic; 290 post-pandemic) and 90 had cancer (16 pre-pandemic; 74 post-pandemic). Post-pandemic, 69% (200) of healthy controls exhibited elevated SARS-CoV-2 seroreactivity, with 51% (148) meeting the criteria for seropositivity. Children with cancer showed similar rates of seroreactivity (67%) and seropositivity (57%) after adjusting for sampling year, with incidence increasing annually from 2020 to 2022. Pre-pandemic samples exhibited weak cross-reactivity to HCoV-OC43 which increased following SARS-CoV-2 infection; however, no significant boosting of HCoV antibodies was observed.

**Conclusions:**

These findings indicate widespread SARS-CoV-2 exposure among children in Western Kenya and provide no evidence that hospitalization heightened infection risk for pediatric patients with cancer.

## Introduction

The COVID-19 pandemic disrupted cancer care globally, with substantial impacts seen in low- and middle-income countries [1,2]. In sub-Saharan Africa, where pediatric cancer survival rates are among the lowest worldwide [3], the pandemic further strained an already fragile healthcare system. Pediatric patients with cancer faced critical delays in care while being treated in hospitals dealing with shortages of diagnostic tests, personal protective equipment, and infrastructure to support physical distancing [4–6]. These suboptimal infection control conditions, combined with the immunocompromised status of patients with cancer, raised concerns about SARS-CoV-2 exposure risk. Yet, infection rates in children with cancer across Africa remain poorly documented.

The paucity of pediatric SARS-CoV-2 infection data is partly due to diagnostic challenges in resource-limited settings, where mild or asymptomatic infections, which are common in children, often go undetected [7,8]. In this context, serological assays have emerged as a critical tool for estimating the true burden of SARS-CoV-2 infection [9,10]. Serosurveys conducted across sub-Saharan Africa have documented widespread infection; however, few studies have included children [11–14], and none have focused on children with cancer.

Despite the utility of serosurveys, accurate interpretation of serological assays remains challenging due to potential cross-reactivity from previous exposure to endemic pathogens, including common human coronaviruses (HCoVs) [15,16]. This issue is particularly pronounced in sub-Saharan Africa, where pre-pandemic cross-reactivity is significantly higher than in other countries and varies by geography and antigenic target [17–19]. Although such cross-reactive responses are speculated to offer partial immune protection, they can also lead to overestimation of SARS-CoV-2 seroprevalence by producing false-positive signals. As a result, the use of multiple SARS-CoV-2 antigens and regional pre-pandemic controls is recommended to help distinguish true infection from background reactivity [20].

Here, we used multiplex antibody profiling to estimate SARS-CoV-2 exposure among pediatric patients with cancer admitted to regional cancer referral hospitals in Western Kenya. All patients were unvaccinated and asymptomatic for COVID-19 at the time of sampling. We compared antibody profiles to healthy community-based controls to determine whether children with cancer experienced disproportionate infection risk in a hospital-based setting. By analyzing pre- and post-pandemic plasma samples and incorporating responses to multiple SARS-CoV-2 antigens, we aimed to generate more accurate estimates of prior infection. In parallel, we profiled responses to common HCoVs to explore potential cross-reactive or cross-protective mechanisms between coronaviruses.

## Materials and methods

### Study participants and sample collection

**Fig 1:**
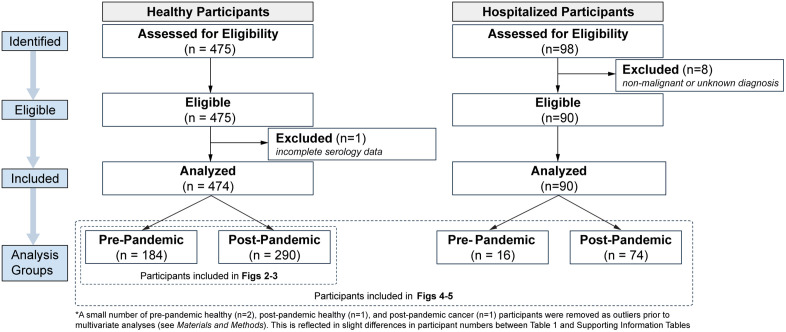
Flow diagram of participant eligibility, group allocation, and inclusion in analyses.

This retrospective case-control study enrolled children under 16 years of age with suspected cancer admitted to Jaramogi Oginga Odinga Teaching and Referral Hospital (JOOTRH) in Kisumu, Kenya, or Moi Teaching and Referral Hospital (MTRH) in Eldoret, Kenya, between March 2014 and October 2022. Confirmation of cancer diagnosis was required for case inclusion (Fig 1; S9-S10 Tables). All patients with cancer were sampled before initiation of treatment. Healthy community controls were recruited from rural hospitals in geographically matched areas of Western Kenya, including Ahero and Chulaimbo in Kisumu County and Mosoriot near Eldoret, which are among the rural facilities that refer cancer patients to JOOTRH and MTRH [21]. None of the participants exhibited symptoms of COVID-19 or received molecular diagnostic testing, as this was not widely available in Kenya during the study period. Vaccination history was recorded at time of blood collection, and all participants were unvaccinated against SARS-CoV-2 at the time of sampling.

Venous blood samples were collected from each participant in sodium heparin vacutainer tubes. Plasma was separated by centrifugation and stored at -20°C until analysis. Each participant contributed a single sample; the dataset is therefore cross-sectional with no repeated measurements. Post-pandemic samples from the cancer cohort were collected between 2020 and 2022, whereas healthy post-pandemic sampling was restricted to 2022 (S16 Fig). Participant characteristics are summarized in Table 1.

**Table 1:** Demographics of study participants.

	All	Healthy		Cancer	
	(n = 564)	Pre-Pandemic (n = 184)	Post-Pandemic (n = 290)	Pre-Pandemic (n = 16)	Post-Pandemic (n = 74)
**Site** (No. (%))					
Chulaimbo	133 (24%)	133 (72%)	··	··	··
Mosoriot	149 (26%)	51 (28%)	98 (34%)	··	··
Ahero	192 (34%)	··	192 (66%)	··	··
MTRH	62 (11%)	··	··	2 (12%)	60 (81%)
JOORTH	28 (5%)	··	··	14 (88%)	14 (19%)
**Age** (Mean (SD))	6.1 (2.7)	7.9 (2.5)	4.5 (1.1)	8.1 (2.8)	7.1 (3.8)
**Sex** = Male (%)	310 (55%)	117 (64%)	140 (48%)	8 (50%)	45 (60%)
**Year Collected** (No. (%))					
≤2018	15 (3%)	··	··	15 (94%)	··
2019	134 (23%)	133 (72%)	··	1 (6%)	··
2020	72 (13%)	51 (28%)	··	··	21 (28%)
2021	22 (4%)	··	··	··	22 (30%)
2022	321 (57%)	··	290 (100%)	··	31 (42%)

This study was approved by the Institutional Review Board at the University of Massachusetts Chan Medical School, Worcester, MA, USA, the Scientific and Ethical Research Unit at the Kenya Medical Research Institute (KEMRI), and the Institutional Research and Ethics Committee at MTRH, Kenya. Enrollment occurred between March 25, 2014 and October 22, 2022. University of Massachusetts Chan Medical School approvals covered all study activities during this period. The Scientific and Ethical Research Unit at KEMRI provided ethical approval for all Kenyan study sites until 2017, at which time the Institutional Research and Ethics Committee at MTRH requested a separate review for children admitted to their hospital and enrolled in this study. Written informed consent was obtained from parents or legal guardians of all minor study participants. For post-pandemic samples, written informed consent was obtained for prospective blood collection and serological testing for studies including malaria, Epstein-Barr virus, and other pathogens of interest, with approval for future use. For pre-pandemic samples, written informed consent allowed sample storage for future pathogen testing, and the samples were retrospectively included in this analysis.

### Luminex profiling

A multiplex Luminex assay was used to measure antigen-specific antibody isotypes, subclasses, and Fcγ receptor (FcγR) binding [22–25]. Antigens (S1 Table) were covalently coupled to MagPlex microspheres following the Luminex xMAP Cookbook. Briefly, magnetic beads were activated with EDC and Sulfo-NHS in 0.1 M monosodium phosphate buffer for 20 minutes at room temperature. After washing with 0.05M MES buffer, beads were incubated with antigen for 2 hours at room temperature, washed with storage buffer (PBS with 0.1% BSA, 0.02% Tween-20, 0.05% sodium azide, pH 7.4), and stored at 4°C until use.

FcγR probes (FcγR2A and FcγR3A) were obtained from the Duke Human Vaccine Institute Protein Production Facility, biotinylated using the BirA biotin-protein ligase kit (Avidity, Cat#BirA500-BirA500), and conjugated to Streptavidin-PE (Agilent, Cat#PJ315-1) as previously described [26,27].

To quantify antibody levels and FcγR binding, antigen-coupled beads were incubated with appropriately diluted plasma samples (S2 Table) for 2 hours at room temperature. After washing, PE-conjugated secondary antibodies or PE-FcγR probes were added and incubated for 1 hour at room temperature. Beads were washed, resuspended in ABE buffer, and fluorescence was acquired on a FlexMap3D (Luminex). Relative antibody levels and FcγR binding is reported as median fluorescence intensity (MFI). Additional assay details are provided in S1 Appendix.

### Statistical analysis

This analysis was restricted to the 564 participants who met inclusion criteria and had complete serology measurements (Fig 1). Participants were classified as pre-pandemic or post-pandemic based on sample collection date, with February 1, 2020 as the cutoff, approximately six weeks prior to the first confirmed COVID-19 case in Kenya on March 12, 2020 (S16 Fig) [28]. Age and sex were evaluated as potential confounders for SARS-CoV-2-specific serologic responses and FcγR binding across study groups; however, no significant associations were observed (S17 Fig). Participants were therefore stratified by health status and sampling period only for all downstream analyses. All analyses were performed in R (v4.5.1).

Serology measurements were background-adjusted by subtracting the mean value of bovine serum albumin wells, and negative values were set to zero. Antibody features with ≥ 25% zero values across all analysis groups (Table 1) were removed from the dataset. Participants with Hotelling’s reduced T² statistics above the 95^th^ percentile of the chi-squared distribution, as determined by principal component analysis (PCA), were classified as outliers and excluded from multivariate analyses (n = 4; Fig 1). Prior to multivariate modelling, data were log-transformed and batch-corrected using ‘ComBat’ (‘sva’ package; v3.56.0).

Hierarchical clustering was applied to SARS-CoV-2-specific antibody features from healthy participants (pre- and post-pandemic) to identify individuals with elevated SARS-CoV-2 seroreactivity profiles relative to pre-pandemic samples. To avoid bias from altered antibody profiles due to B cell malignancies, which accounted for the majority (71% [64 of 90]) of cancer cases in our cohort (S9-S10 Tables), clustering excluded patients with cancer. Data were mean-centered and variance-scaled prior to clustering, which was performed using complete linkage and Euclidean distance with the ‘pheatmap’ function (‘pheatmap’ package; v1.0.13). The optimal cluster number was selected via the elbow method (S1 Fig).

To extend the phenotypes identified in healthy controls to patients with cancer, we trained a k-nearest neighbors (k-NN) classifier (k = 7) using the ‘class’ package (v7.3.23) on the first three principal components derived from PCA-reduced SARS-CoV-2-specific antibody features of healthy post-pandemic controls. Seroreactivity phenotype served as the outcome label. We then applied the trained classifier to PCA-transformed antibody features from cancer participants to predict their seroreactivity phenotypes. Model performance was evaluated using five-fold cross-validation and compared to a null distribution generated from 1000 label-permuted models. Statistical significance was assessed via tail probability (S1 Appendix).

To differentiate true SARS-CoV-2 infection from cross-reactive responses, we applied a multi-antigen serology approach leveraging differences in sequence conservation and antibody kinetics between nucleocapsid (N) and receptor-binding domain (RBD) proteins. The N protein is relatively conserved across human coronaviruses, making it more prone to cross-reactive binding [16–18,29,30], whereas the RBD is more specific to SARS-CoV-2 infection [31,32] and induces longer-lasting SARS-CoV-2-specific antibodies [33,34], serving as a more reliable marker of prior infection. Accordingly, RBD seropositivity was used as the primary indicator of prior SARS-CoV-2 infection, with interpretation of N seropositivity conditional on RBD status.

Based on this framework, samples were classified into four exposure groups. Individuals seropositive for both N and RBD were classified as ‘recent infection’, reflecting detectable responses to both antigens. Individuals seropositive for RBD alone were classified as ‘remote infection’, consistent with the more rapid waning of anti-N responses relative to anti-RBD following SARS-CoV-2 infection [33,34]. Individuals seropositive for N alone were classified as ‘cross-reactive’. Although anti-N responses wane more quickly than other antigens following SARS-CoV-2 infection, endemic HCoVs circulate widely and reinfect individuals repeatedly, periodically boosting cross-reactive N antibodies independent of SARS-CoV-2 exposure. Isolated N reactivity in the absence of RBD seropositivity is therefore more consistent with endemic exposure with common HCoVs than prior SARS-CoV-2 infection [16–18,29,30]. Individuals seronegative for both were classified as ‘non-reactive’.

Seropositivity was defined as total IgG levels exceeding the 95^th^ percentile of all healthy pre-pandemic samples (collected before February 1, 2020). To account for the possibility that samples collected in early 2020 (prior to February) may capture undetected early SARS-CoV-2 circulation, sensitivity analyses were performed using only pre-pandemic samples collected before January 1, 2020. Results were consistent with the primary analysis; therefore, all pre-pandemic samples were retained for the final analyses (S2 Fig).

To assess cross-reactivity between common HCoVs and SARS-CoV-2, we stratified post-pandemic participants by their exposure status. Participants classified as cross-reactive or non-reactive were considered ‘unexposed’, and those classified as recent or remotely infected were considered ‘exposed’. Partial Spearman correlations, controlling for study group, were calculated between anti-spike IgG levels to HCoVs (HCoV-229E, HCoV-NL63, HCoV-HKU1, HCoV-OC43) and SARS-CoV-2 spike protein. Multiple comparisons were corrected using the Benjamini-Hochberg method. Differences in HCoV antibody levels between exposure status were tested to explore cross-boosting, using the Kruskal-Wallis test with Benjamini-Hochberg correction, followed by Dunn’s post-hoc test when significant. Statistical significance was defined as p < 0.05.

Differences in seroreactivity phenotype and exposure group distributions between healthy participants and participants with cancer were assessed using two-proportion z-tests.

## Results

We profiled antibody responses to SARS-CoV-2 and common HCoVs in plasma samples from 564 children recruited between 2014 and 2022 at regional cancer referral hospitals (JOOTRH and MTRH), rural county hospitals (Ahero), and sub-county hospitals (Chulaimbo and Mosoriot) in Western Kenya. The cohort had a mean age of 6.1 years (SD 2.7), and 55% (310 of 564) were male. At the time of sampling, all children were unvaccinated against SARS-CoV-2, asymptomatic for COVID-19, and had unknown SARS-CoV-2 infection histories. Of the 564 participants, 474 were healthy and 90 had a cancer diagnosis. To assess the relative impact of the COVID-19 pandemic on hospitalized patients with cancer, participants were stratified by health status (healthy vs cancer) and sampling period (pre- vs post-pandemic). Demographic characteristics for the analysis groups are summarized in Table 1.

**Fig 2:**
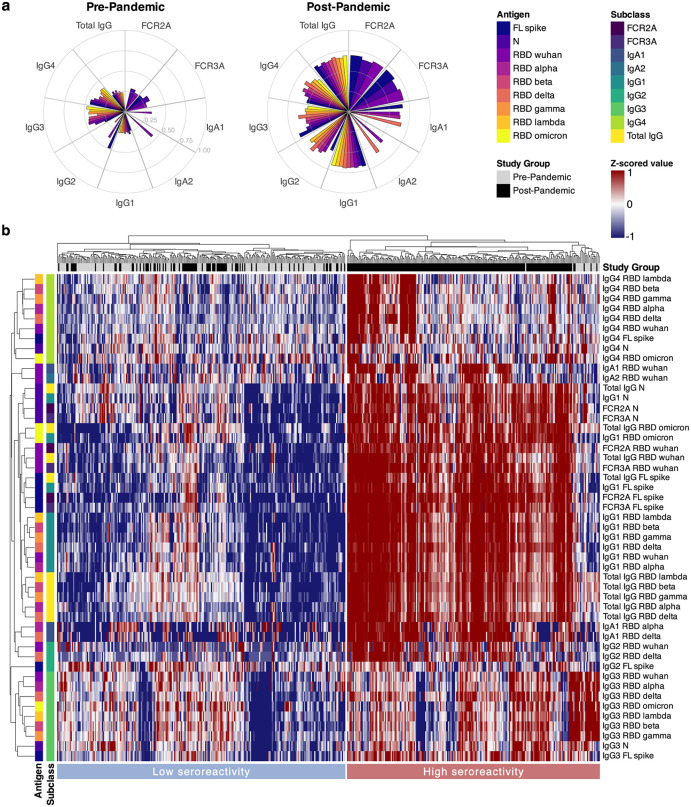
Serological profiling reveals increased SARS-CoV-2 seroreactivity in healthy children sampled post-pandemic compared to pre-pandemic controls. **(a)** Polar plots show the median percentile ranks of the antibody features measured within plasma samples from healthy children sampled before and after the onset of the COVID-19 pandemic. Each wedge represents an antibody feature, and the size of the wedge depicts the median percentile ranging from 0 to 1. **(b)** Hierarchical clustering of z-scored antibody features distinguishes pre- and post-pandemic samples, revealing two dominant seroreactivity phenotypes characterized by differentially low (left cluster; dark blue) and high (right cluster; dark red) SARS-CoV-2 seroreactivity. N: Nucleocapsid, RBD: Receptor binding domain, MFI: mean fluorescence intensity.

To minimize confounding from cancer-related immune modulation, we first analyzed SARS-CoV-2-specific antibody responses in healthy children sampled pre- and post-pandemic. Post-pandemic participants exhibited significantly higher SARS-CoV-2-specific antibody levels and FcγR binding compared to pre-pandemic participants, despite all participants being asymptomatic (Fig 2a; S3 Fig).

We then performed hierarchical clustering on SARS-CoV-2 antibody features to identify immune response phenotypes associated with the elevated antibody levels observed. Clustering identified two distinct phenotypes: a ‘high seroreactivity’ group with broadly elevated SARS-CoV-2 antibody binding and FcγR engagement suggestive of prior exposure or cross-reactivity, and a ‘low seroreactivity’ group resembling pre-pandemic profiles (Fig 2b). Among the 289 post-pandemic participants, 200 (69%) clustered in the high seroreactivity group and 89 (31%) in the low seroreactivity group. Post-pandemic participants in the high seroreactivity cluster were less likely to be from Ahero, compared to those with low seroreactivity (p = 0.02; S3 Table).

Within the high seroreactivity cluster, 62 (31%) post-pandemic samples exhibited relatively elevated SARS-CoV-2-specific IgG4 responses. This IgG4-high subgroup also showed broadly elevated IgG4 responses to multiple endemic HCoV antigens (S18 Fig), and IgG4 levels against HCoV and SARS-CoV-2 spike antigens were positively correlated across the post-pandemic cohort (S19 Fig). Importantly, a similar correlation pattern between HCoV- and SARS-CoV-2-specific IgG4 responses was also observed in pre-pandemic, SARS-CoV-2-naïve individuals (S19 Fig). These findings suggest that elevated SARS-CoV-2 IgG4 responses in this subgroup may reflect pre-existing cross-reactive antibody patterns shaped by prior HCoV exposure rather than de novo SARS-CoV-2 infection-driven immunity.

Notably, 20 (11%) of the 182 pre-pandemic samples also clustered within the high seroreactivity group. Within the pre-pandemic cohort, children in the high seroreactivity cluster were older than those in the low seroreactivity cluster (p = 0.04; S3 Table) and exhibited broadly elevated IgG and IgG3 levels, as well as increased FcγR binding (S4-S5 Fig). In addition, we observed significant positive correlations between age and HCoV-specific IgG and IgG3 responses in pre-pandemic samples (S20 Fig), suggesting that the elevated IgG3 responses in this subgroup may reflect progressively increasing cumulative HCoV exposure with age, leading to enhanced cross-reactive antibody responses [35].

**Fig 3:**
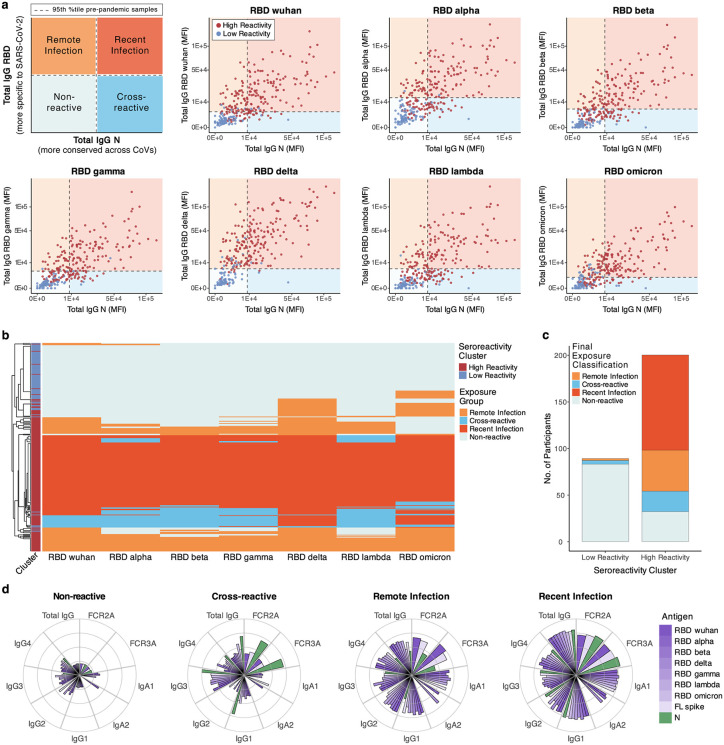
Elevated SARS-CoV-2 seroreactivity observed in children sampled post-pandemic likely reflects past infection. **(a)** Scatter plots display total IgG antibody levels against N and RBD for seven RBD variants in post-pandemic healthy children. Points are colored by seroreactivity phenotype cluster assignment from Fig 2b (high: dark red; low: dark blue). Dashed lines indicate seropositivity thresholds, defined as the 95^th^ percentile of healthy pre-pandemic samples. Samples seropositive for both N and RBD were classified as ‘recent infection’ (red); those exceeding RBD only as ‘remote infection’ (orange); N only as ‘cross-reactive’ (light blue); and samples below both thresholds as ‘non-reactive’ (grey); as depicted in the schematic in the upper-left. **(b)** Hierarchical clustering of exposure group assignment across RBD variants for each post-pandemic sample. **(c)** Stacked bar chart showing the distribution of samples across exposure classifications stratified by seroreactivity phenotype. Final exposure classification was based on the majority label assigned across all RBD variants. **(d)** Polar plots depict the median percentile rank (0-1) of antibody features in post-pandemic samples grouped by final exposure classification.

To clarify whether elevated seroreactivity in healthy post-pandemic participants reflected prior SARS-CoV-2 infection or cross-reactivity due to endemic exposure with common HCoVs, we classified participants into four exposure groups based on total IgG levels to N and RBD antigens: ‘recent infection’ (seropositive for N and RBD), ‘remote infection’ (seropositive for RBD), ‘cross-reactive’ (seropositive for N), and ‘non-reactive’ (seronegative for both) [16–18,29–34]. Classification was performed for each of the seven RBD variants assayed, and final assignment was based on a majority vote (Fig 3a–3c). This approach demonstrated high specificity when applied to pre-pandemic samples with and without cancer, correctly classifying 96% (191 of 198) as non-reactive or cross-reactive (S6 Fig).

Applying the multi-antigen classification scheme to the 289 healthy post-pandemic participants, we identified 102 (35%) as recently infected, 46 (16%) as remotely infected, 26 (9%) as cross-reactive, and 115 (40%) as non-reactive, resulting in an estimated SARS-CoV-2 exposure rate of 51%. The majority (146 of 200 [73%]) of post-pandemic participants in the high seroreactivity cluster were classified as recently or remotely infected; however, 54 (27%) were classified as cross-reactive or non-reactive, indicating that some participants with elevated antibody responses did not meet serological criteria for SARS-CoV-2 exposure. Infection rates were significantly lower in Ahero (p < 0.001), but other demographic factors were balanced between groups (S4 Table).

Evaluation of broader seroprofiles across exposure groups revealed consistent antibody binding specificity to spike (RBD and full-length) and nucleocapsid antigens across IgG subclasses and FcγRs, especially IgG1, FcγR2A, and FcγR3A (Fig 3d). This coordinated immune signature supports the robustness of our serological classification and highlights distinct immunological responses between exposure groups beyond IgG levels.

**Fig 4:**
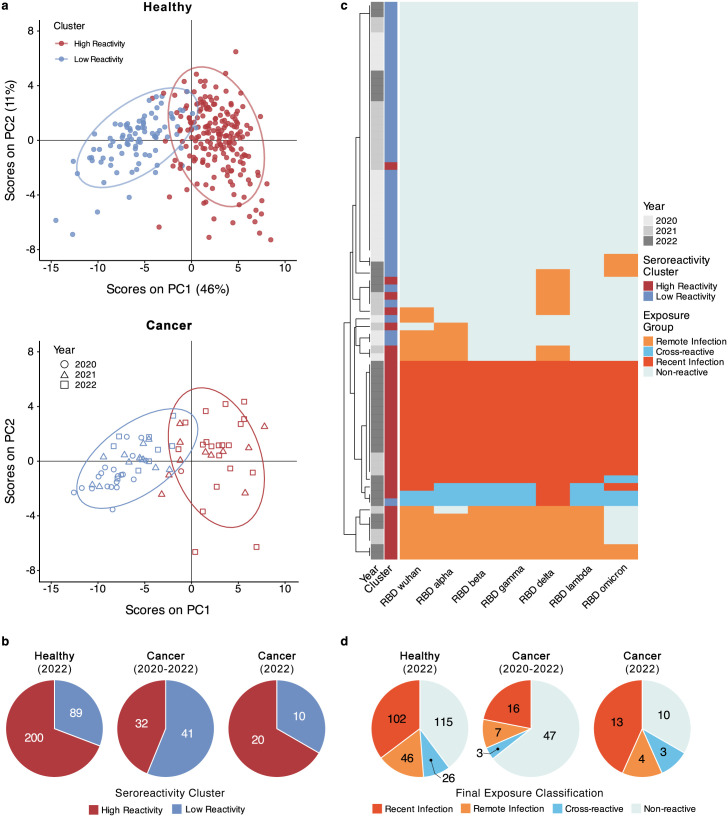
Predicted SARS-CoV-2 seroreactivity and exposure rates in children with cancer are similar to healthy community-based controls after adjusting for sampling year. **(a)** In the upper panel, SARS-CoV-2 serology data from healthy post-pandemic controls was dimensionally reduced by PCA, and samples were plotted according to their scores on the first two principal components. Ellipses represent the 95% confidence regions for scores in each group. A k-NN model trained on the first three principal components of the reduced dataset classified healthy samples into high or low seroreactivity clusters with 94% accuracy (96% sensitivity, 91% specificity). In the lower plot, antibody profiles from children with cancer were projected onto the same PCA space and seroreactivity phenotypes for these samples were predicted using the k-NN classifier developed on healthy children. Points are colored by seroreactivity phenotype (high: dark red; low: dark blue) and shaped by collection year (2020: circles; 2021: triangles; 2022: squares). **(b)** Pie charts compare the distribution of seroreactivity phenotypes among all post-pandemic cancer samples (middle) and the subset collected in 2022 (right), alongside healthy controls (left). **(c)** Hierarchical clustering of exposure groups for post-pandemic participants with cancer across RBD variants, using the classification schema outlined in Fig 3. **(d)** Pie charts show the distribution of final exposure classifications in post-pandemic patients with cancer relative to healthy controls. PCA: principal component analysis; PC: principal component; k-NN: k-nearest neighbor.

To extend the seroreactivity phenotypes identified in healthy controls to patients with cancer we trained a k-NN model using SARS-CoV-2-specific antibody features from healthy post-pandemic controls to predict seroreactivity phenotypes (Fig 4a). The model distinguished high and low seroreactivity phenotypes in the training (i.e., healthy) dataset with 96% sensitivity and 91% specificity in cross-validated analyses, significantly outperforming 1000 label-permuted classifiers (p < 0.001; S7 Fig). When applied to the 73 post-pandemic patients with cancer, the k-NN model classified 32 (44%) as high seroreactive and 41 (56%) as low seroreactive (Fig 4a–4b).

Using the same multi-antigen classification approach applied to healthy participants, analysis of anti-N and anti-RBD IgG levels in post-pandemic patients with cancer estimated a SARS-CoV-2 exposure rate of 32% (23 of 73). Among these, 16 (22%) were classified as recently infected, 7 (10%) as remotely infected, 3 (4%) as cross-reactive, and 47 (64%) as non-reactive (Fig 4c–4d; S8 Fig). Although a higher proportion of patients from MTRH were classified in the high-reactivity group compared to JOORTH, site was not significantly associated with exposure classification (S5-S6 Tables), suggesting this difference does not reflect meaningful variation in infection risk. By contrast, seroprevalence increased markedly over time, from 0% in 2020 to 27% in 2021 and 57% in 2022 (p < 0.001; S9 Fig; S5-S6 Tables). To account for this temporal trend and better align with the dates of healthy control sampling (S16 Fig), subsequent analyses were restricted to patients with cancer sampled in 2022.

Within this subset of 30 patients with cancer, 67% (20) demonstrated high seroreactivity (Fig 4b). Of these, 57% (17) showed evidence of SARS-CoV-2 exposure, with 13 (44%) classified as recently infected, 4 (13%) as remotely infected, 3 (10%) as cross-reactive, and 10 (33%) as non-reactive (Fig 4c–4d). These rates of exposure and seroreactivity were similar to those observed in healthy controls sampled during the same year (exposure, p = 0.71; seroreactivity, p = 0.93). No demographic differences were detected between patients with cancer sampled in 2022 when stratified by seroreactivity cluster or exposure group (S7-S8 Tables).

**Fig 5:**
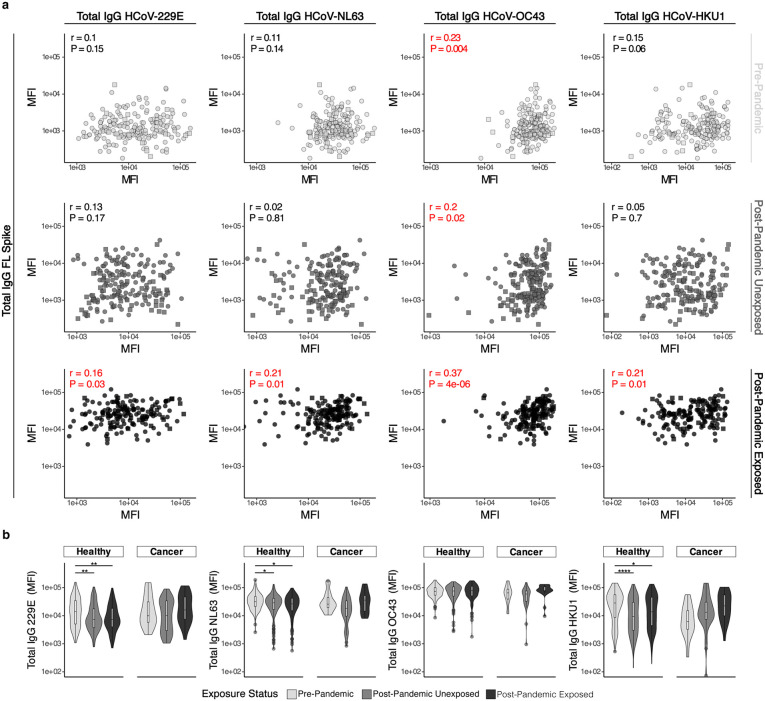
SARS-CoV-2 exposure modestly enhances IgG cross-reactivity between SARS-CoV-2 and common HCoVs, despite comparable HCoV antibody levels. **(a)** Correlation between total IgG to the SARS-CoV-2 full-length (FL) spike (y-axis) and to FL-spike protein on common HCoVs (229E, NL63, OC43, HKU1; x-axis) in healthy children (circles) and children with cancer (squares). Points are colored by sampling period: pre-pandemic (light grey), post-pandemic unexposed (dark grey), or post-pandemic exposed (black). Post-pandemic exposure status was defined from Figs 3-4, with recent infection or past infection considered exposed and non-reactive or cross-reactive considered unexposed. Partial Spearman correlation coefficients (r) and p-values are shown, controlling for study group. P-values were adjusted for multiple comparisons, and significant correlations are denoted by red text. **(b)** Univariate plots showing total IgG levels against common HCoVs for healthy children (left) and children with cancer (right) stratified by exposure status. Statistical significance was assessed using the Kruskal-Wallis test with Benjamini-Hochberg correction for multiple comparisons. Dunn’s post-hoc test was performed for significant results (*p < 0.05, **p < 0.01, ***p < 0.001, ****p < 0.0001). HCoVs: human coronaviruses; FL Spike: full-length spike.

Finally, to assess potential cross-reactivity between SARS-CoV-2 and common HCoVs, we correlated total IgG levels to the SARS-CoV-2 spike protein with responses to spike proteins from HCoV-229E, HCoV-NL63, HCoV-OC43, and HCoV-HKU1. In pre-pandemic samples, there were weak correlations between SARS-CoV-2 and the betacoronaviruses (HCoV-OC43, HCoV-HKU1), with a significant association observed for HCoV-OC43 (r = 0.23, p = 0.004; Fig 5a). A similar correlation with HCoV-OC43 was found in post-pandemic participants without evidence of SARS-CoV-2 exposure (r = 0.2, p = 0.02). In contrast, SARS-CoV-2-exposed post-pandemic participants showed moderately stronger correlations across all HCoVs, particularly for HCoV-OC43 (r = 0.37, p = 4E-6). However, no significant increases in anti-HCoV IgG levels were observed between post-pandemic participants based on exposure group, infection status, or seroreactivity phenotype (Fig 5b; S10-S12 Figs). Similar trends were also observed for other IgG subclasses and antibody isotypes (S10-S12 Figs), suggesting modest cross-reactivity between SARS-CoV-2 and HCoV-OC43 without evidence of cross-boosting.

## Discussion

This study estimates SARS-CoV-2 seroprevalence in hospitalized children with cancer and healthy community controls in Kenya during the COVID-19 pandemic. To our knowledge, it is the first study to report seroprevalence estimates in a pediatric oncology cohort in Kenya or elsewhere in Africa. By October 2022, we found that 57% of children with cancer and 51% of controls had SARS-CoV-2 antibodies indicative of prior infection. Importantly, despite widespread exposure, our findings suggest that hospital-based oncology care in rural Kenya did not significantly increase infection risk among pediatric patients with cancer.

While few seroprevalence studies have been conducted on children with cancer, and none in low- or middle-income countries, our results are consistent with evidence from high-income settings, where children with cancer have shown comparable SARS-CoV-2 infection rates to healthy children [36,37]. Notably, this result contrasts with data from adult oncology populations in Tanzania, which reported that adults with cancer had lower SARS-CoV-2 seroprevalence compared to blood donors [38], a difference that may reflect age-related susceptibility or behavioral factors such as adherence to isolation measures [39]. The lower seroprevalence observed in adult oncology patients in Tanzania may also reflect attenuated seroconversion due to impaired humoral immunity in cancer patients [38,40]. Importantly, humoral impairment is unlikely to be a major contributor to serological differences in our cohort, as all patients were sampled before chemotherapy initiation and malignancy alone appears to have limited effects on SARS-CoV-2 seroconversion [41,42]. Consistent with this, antibody profiles were broadly similar between healthy controls and cancer patients across the pre- and post-pandemic periods (S21 Fig). While additional studies are needed to determine whether these trends extend to earlier pandemic stages or other immunocompromised groups, our findings provide encouraging early evidence supporting the effectiveness of hospital-level infection control measures during the COVID-19 pandemic in a resource-limited setting.

Our seroprevalence estimates indicate widespread SARS-CoV-2 transmission among children in rural Kenya, with increasing incidence over the course of the pandemic. Although data spanning multiple years were only available for children with cancer, we observed a clear rise in seropositivity from 2020 to 2022. These temporal trends align with reports across Kenya [43,44] and Africa [19] and are likely attributable to the combined effects of easing government-imposed travel restrictions, reopening of schools, and the new emergence of variants of concern [45,46]. In Kenya specifically, the Delta variant became predominant in mid-2021, followed by the emergence and rapid spread of Omicron in late 2021 and through 2022 [47,48], suggesting that successive infection waves dominated by high transmissible variants were likely key drivers of transmission in this cohort.

By 2022, seroprevalence in both cancer patients and healthy controls approached 60%, closely matching the 62% reported in Kilifi, another rural county in Kenya [44]. In contrast, urban centers such as Nairobi reported seroprevalence exceeding 80% during the same period [44], reflecting a well-documented urban-rural disparity across sub-Saharan Africa likely driven by differences in population density [19]. However, population density alone could not explain the variation we observed within rural areas. Participants from Ahero exhibited lower seroprevalence than those from Mosoriot, despite Ahero’s marginally higher population. This discrepancy may instead reflect variation in community-level health awareness, shaped by local involvement in research and public health initiatives. Ahero is a long-standing clinical trial site where many children have participated in large malaria vaccine studies, potentially increasing community familiarity with infectious disease prevention measures. By comparison, Mosoriot has had limited research engagement, and its residents may have had reduced awareness or adoption of COVID-19 mitigation strategies. These findings highlight that even within rural areas, differences in local health literacy and engagement may influence patterns of SARS-CoV-2 transmission.

We also explored whether pre-existing immunity from common HCoVs could influence SARS-CoV-2 antibody responses. Our analysis of common HCoVs revealed limited antibody cross-reactivity with SARS-CoV-2 that appears insufficient to confer cross-protective immunity. Notably, antibody responses to SARS-CoV-2 correlated significantly with those against the betacoronavirus HCoV-OC43, consistent with HCoV-OC43’s higher sequence homology to SARS-CoV-2 compared to other HCoVs [29]. Weak correlations with HCoV-OC43 were observed in both pre-pandemic and SARS-CoV-2 naïve post-pandemic samples. These correlations strengthened following SARS-CoV-2 exposure. However, we found no corresponding rise in HCoV antibody levels, indicating minimal cross-boosting and suggesting that prior HCoV exposure may have limited influence on effective SARS-CoV-2 immunity. These results are consistent with previous studies demonstrating limited cross-neutralization by pre-pandemic sera [16,49,50]. Nevertheless, the presence of any cross-reactive behavior, particularly in pre-pandemic samples, reinforces the importance of distinguishing cross-reactivity from virus-specific responses when interpreting serological data.

To this end, a major strength of our study is the use of a multi-antigen serological approach combined with regionally matched pre-pandemic controls to define seropositivity thresholds. This approach improves the accuracy of seroprevalence estimates in contexts where antibody cross-reactivity is common and provides a practical framework for improving specificity in SARS-CoV-2 serosurveillance, particularly in distinguishing responses from other human coronaviruses (HCoVs, MERS, SARS-CoV-1). Residual cross-reactivity from other endemic exposures, including malaria, may persist at low levels but is unlikely to materially affect overall estimates (S13 Fig) [51].

This study has limitations. First, our classification approach focuses on IgG responses, which may miss early infections dominated by IgM and does not capture other features of humoral immunity such as affinity, avidity, or antibody kinetics. However, analyses of paired IgM measurements indicated minimal contribution of early IgM responses to overall seropositivity estimates (S14 Fig). Additionally, our exposure classification depends on antigen-defined seroconversion patterns, including RBD- and N-based responses, and assumes cross-reactive responses across SARS-CoV-2 variants [52–55]. Although some individuals may exhibit N-only seroconversion [56] or weaker responses to specific variants [57,58], sensitivity analyses using alternative antigen combinations and more permissive classification schemes yielded similar exposure estimates and did not meaningfully change the study conclusions (S15 Fig; S22 Fig). Nevertheless, serological profiles cannot establish infection timing or exposure history with certainty. For example, it is possible that cross-reactive boosting of anti-N antibodies by endemic HCoVs may contribute to some N+/RBD+ profiles, and antibody waning may result in misclassification of previously infected individuals as seronegative. Our classifications should therefore be interpreted as serological approximations rather than definitive reconstructions of infection history. Lastly, the absence of temporal data from healthy post-pandemic participants in our cohort limited our ability to fully characterize infection risks between children with and without cancer before 2022. However, prior serosurveys conducted in Kisumu County, our primary sampling site, reported 30% seroprevalence in 2021 [43], closely matching the 27% estimate from our cancer cohort that year, suggesting that our findings may be generalizable to earlier pandemic stages. Nonetheless, future studies are needed to explore temporal trends and evolving risks more comprehensively.

In summary, our findings provide early but encouraging evidence that pediatric cancer care in African settings can be delivered safely without significantly increasing SARS-CoV-2 exposure risk. These insights have important implications for maintaining continuity of cancer care during pandemics, particularly in limited resource settings where healthcare disruptions can have disproportionate consequences.

## Supporting information

S1 Appendix Supplementary methods. (PDF)

S1 Data Serology data and associated metadata. (XLSX)

S1 Table Antigens in Luminex assay. (PDF)

S2 Table Antibodies and FcRs in Luminex assay. (PDF)

S3 Table Demographics of healthy participants by seroreactivity phenotype. (PDF)

S4 Table Demographics of healthy post-pandemic participants by estimated exposure groups. (PDF)

S5 Table Demographics of post-pandemic cancer patients by seroreactivity phenotype. (PDF)

S6 Table Demographics of post-pandemic cancer patients by estimated exposure groups. (PDF)

S7 Table Demographics of cancer patients sampled in 2022 by seroreactivity phenotype. (PDF)

S8 Table Demographics of cancer patients sampled in 2022 by estimated exposure groups. (PDF)

S9 Table Summary of cancer diagnoses in pre-pandemic samples. (PDF)

S10 Table Summary of cancer diagnoses in post-pandemic samples. (PDF)

S1 Fig Determination of optimal cluster number. Elbow plot depicting the within-cluster sum of squares versus the number of clusters. The optimal cluster number for hierarchical clustering was determined by the inflection point, denoted by the dashed line.
(PDF)

S2 Fig Exposure classifications are robust to the choice of pre-pandemic reference population. Pie charts depict final exposure classifications using seroreactivity thresholds derived from either all pre-pandemic healthy samples (top row) or only those collected before January 1, 2020 (bottom row). Results were highly consistent across definitions, with no evidence of differences in estimated exposure between healthy children and children with cancer sampled in 2022 using Fisher’s exact test (including Jan. 2020 samples: p = 0.83; excluding Jan. 2020 samples: p = 0.73). Estimates in the cancer cohort remained unchanged across thresholds.
(PDF)

S3 Fig SARS-CoV-2 antibody levels in healthy children. SARS-CoV-2-specific antibody levels in healthy children sampled pre-pandemic (light grey; n = 184) and post-pandemic (dark grey; n = 290). Statistical significance was assessed using the Mann-Whitney U test, with multiple comparisons adjusted by the Benjamini-Hochberg procedure (*p < 0.05, **p < 0.01, ***p < 0.001). N: nucleocapsid, RBD: receptor binding domain, MFI: mean fluorescence intensity.
(PDF)

S4 Fig SARS-CoV-2 antibody levels by seroreactivity cluster in pre-pandemic healthy kids. Comparisons of SARS-CoV-2-specific antibody levels in healthy children sampled pre-pandemic who clustered in the high (red; n = 20) and low (blue; n = 162) seroreactivity groups (as in Fig 2b). Statistical significance was assessed using the Mann-Whitney U test, with multiple comparisons adjusted by the Benjamini-Hochberg procedure (*p < 0.05, **p < 0.01, ***p < 0.001). N: nucleocapsid, RBD: receptor binding domain, MFI: mean fluorescence intensity.
(PDF)

S5 Fig SARS-CoV-2 antibody profiles in pre-pandemic healthy kids by seroreactivity cluster. Polar plots depict the median percentile rank for each SARS-CoV-2 antibody feature from plasma samples from pre-pandemic healthy children who clustered in the low (left; n = 162) and high (right; n = 20) seroreactivity clusters (as in Fig 2b). Each wedge represents an antibody feature, and the size of the wedge depicts the median percentile ranging from 0 to 1.
(PDF)

S6 Fig SARS-CoV-2 exposure classifications in pre-pandemic children. **(a)** Scatter plots of anti-N and anti-RBD antibody levels for seven RBD variants measured in pre-pandemic children with (purple) and without (green) cancer. Dashed lines represent the 95^th^ percentile of healthy pre-pandemic samples, defining quadrants as shown in the upper-left cartoon. **(b)** Hierarchical clustering of exposure classifications for pre-pandemic samples across RBD variants. Final classifications for pre-pandemic samples were: 91% non-reactive (181/198), 5% cross-reactive (10/198), and 4% remote infection (7/198). N: nucleocapsid, RBD: receptor binding domain, MFI: mean fluorescence intensity.
(PDF)

S7 Fig Performance evaluation of k-NN model. **(a)** Classification boundaries generated using k-nearest neighbors (k-NN) to distinguish samples with low (blue) and high (red) SARS-CoV-2 seroreactivity. The k-NN model was trained on the first three principal components of PCA-transformed serology data from healthy children sampled post-pandemic. Data points are colored based on hierarchical clustering classification (as in Fig 2b), while background shading indicates k-NN decision boundaries. **(b)** Performance of the k-NN model in classifying seroreactivity phenotypes among healthy post-pandemic samples when evaluated by 5-fold cross-validation. The model achieved 94% accuracy, 96% sensitivity, and 91% specificity. These values are shown relative to the cross-validated accuracy from 1000 label-permuted datasets. P-values were derived from the tail probability of the null distribution. PC: principal component.
(PDF)

S8 Fig Anti-RBD vs. N plots for children with cancer sampled post-pandemic. Scatter plots of Total IgG N and RBD antibody levels for seven RBD variants measured in post-pandemic children with cancer, colored by SARS-CoV-2 reactivity phenotypes (as in Fig 2b). Dashed lines represent the 95^th^ percentile of healthy pre-pandemic samples, defining quadrants according to the color scheme in the upper left cartoon. Post-pandemic samples exceeding the 95th percentile of pre-pandemic controls for both N and RBD were classified as ‘recent infection’ (red). Samples exceeding the RBD threshold but not N were labeled as ‘remote infection’ (orange), while those exceeding the N threshold but not RBD were considered ‘cross-reactive’ (light blue). Samples below both thresholds were classified as ‘non-reactive’ (grey) for SARS-CoV-2 exposure. N: nucleocapsid, RBD: receptor binding domain, MFI: mean fluorescence intensity.
(PDF)

S9 Fig Seroreactivity and exposure classifications by year. Stacked bar plots depicting the number of post-pandemic children with cancer classified into each seroreactivity phenotype **(a)** and SARS-CoV-2 exposure classification **(b)** by year of sample collection. kNN: k-nearest neighbor.
(PDF)

S10 Fig HCoV antibody levels in children with and without cancer by exposure type. HCoV-specific antibody levels in healthy children (left) and children with cancer (right), grouped by exposure type. Post-pandemic samples were categorized as exposed (recent or past infection) or unexposed (non-reactive or cross-reactive) based on final exposure classification (as in Fig 5). Statistical significance was assessed using the Kruskal-Wallis test with Benjamini-Hochberg correction for multiple comparisons. Dunn’s post hoc test was applied to significant results (*p < 0.05, **p < 0.01, ***p < 0.001, ****p < 0.0001). MFI: mean fluorescence intensity.
(PDF)

S11 Fig HCoV antibody levels in children with and without cancer by exposure classification. HCoV specific antibody levels from healthy children (left) and children with cancer (right) based on final exposure classification. Statistical significance was assessed using the Kruskal-Wallis test with Benjamini-Hochberg correction for multiple comparisons. Dunn’s post hoc test was applied to significant results (*p < 0.05, **p < 0.01, ***p < 0.001, ****p < 0.0001). MFI: mean fluorescence intensity.
(PDF)

S12 Fig HCoV antibody levels in children with and without cancer by seroreactivity phenotype. HCoV specific antibody levels from healthy children (left) and children with cancer (right) based on seroreactivity phenotype. Statistical significance was assessed using the Kruskal-Wallis test with Benjamini-Hochberg correction for multiple comparisons. Dunn’s post hoc test was applied to significant results (*p < 0.05, **p < 0.01, ***p < 0.001, ****p < 0.0001). MFI: mean fluorescence intensity.
(PDF)

S13 Fig Malaria does not appear to significantly impact SARS-CoV-2 antibody profiles. Comparison of Total IgG AMA1 and HRPII levels in healthy, post-pandemic children from Mosoriot **(a)** and Ahero **(b)** stratified by seroreactivity clusters (as in Fig 2b). AMA1 is a surrogate to indicate whether an individual was ever infected with malaria, and HRPII is a surrogate of recent infection. Statistical significance was assessed using the Mann-Whitney U test, with multiple comparisons adjusted by the Benjamini-Hochberg procedure. MFI: mean fluorescence intensity.
(PDF)

S14 Fig SARS-CoV-2 exposure classifications using IgM. **(a**) Scatter plots of IgM N and RBD antibody levels for each RBD variant measured in post-pandemic healthy children from high (dark red) and low (dark blue) SARS-CoV-2 seroreactivity clusters (as in Fig 2b). Dashed lines represent the 95^th^ percentile of healthy pre-pandemic samples, defining quadrants as shown in the upper-left cartoon. **(b)** Hierarchical clustering of exposure classifications for post-pandemic samples across RBD variants. **(c)** Stacked bar chart showing the number of samples classified in each exposure group, stratified by seroreactivity phenotype. Final exposure classifications were based on a majority vote across all RBD variants. Final classifications for samples were: 96% negative (277/289), 3.8% cross-reactive (11/289), < 1% likely infected (1/289). N: nucleocapsid, RBD: receptor binding domain, MFI: mean fluorescence intensity.
(PDF)

S15 Fig SARS-CoV-2 exposure estimates incorporating N-only responses. Pie charts depict exposure estimates for participants when seropositivity is determined using RBD approach as in original analysis (top row) compared to a RBD or N approach (bottom row). P-values from two-proportion z-tests between exposure estimates from healthy children and children with cancer sampled in 2022 were p = 0.71 and p = 0.62 for the top and bottom row, respectively. In the bottom row, participants classified as ‘cross-reactive’ in our original analysis (i.e., those with N-only seropositivity) are now included in the ‘exposed’ group rather than the ‘unexposed’ group.
(PDF)

S16 Fig Distribution of sample collection dates among study participants. Histograms show dates of plasma sample collection for healthy participants (top row) and participants with cancer (bottom row). The red dashed line indicates the cutoff used to define pre- versus post-pandemic periods (February 1, 2020) and the dotted black line represents the first confirmed case of COVID-19 in Kenya (March 12, 2020).
(PDF)

S17 Fig Age and sex exhibit limited influence on SARS-CoV-2 serology levels pre- and post-pandemic. The heatmaps depicts pairwise correlations between age or sex and SARS-CoV-2-specific antibody levels in pre-pandemic healthy **(a)**, pre-pandemic cancer **(b)**, post-pandemic healthy **(c)**, and post-pandemic cancer **(d)** participants. Correlations between serology measurements and age or sex were determined by spearman and pearson correlations, respectively. Color and coefficient reflect the direction and magnitude of each pairwise correlation. Significant correlations after Benjamini-Hochberg adjustment for multiple comparisons are denoted by asterisks. (*p < 0.05). N: nucleocapsid, RBD: receptor binding domain, FL: full length.
(PDF)

S18 Fig HCoV antibody levels in healthy post-pandemic children by IgG4 reactivity subgroup. HCoV-specific antibody levels in healthy children sampled post-pandemic with relative higher (green; n = 62) or lower (purple; n = 138) SARS-CoV-2-specific IgG4 levels. Statistical significance was assessed using the Mann-Whitney U test, with multiple comparisons adjusted by the Benjamini-Hochberg procedure (*p < 0.05, **p < 0.01, ***p < 0.001). Plots include only healthy post-pandemic participants deemed high seroreactive (n = 200). MFI: mean fluorescence intensity.
(PDF)

S19 Fig IgG4 responses to HCoV spike proteins are correlated with increased IgG4 responses to SARS-CoV-2 spike protein. The matrix depicts pairwise Spearman correlations between antibody levels to HCoV and IgG4 response to SARS-CoV-2 spike antigen in healthy post-pandemic **(a)** and pre-pandemic **(b)** controls. Only correlations with p < 0.1 after Benjamini-Hochberg adjustment for multiple comparisons are shown. Color and coefficient reflect the direction and magnitude of each pairwise correlation. N: nucleocapsid, RBD: receptor binding domain, FL: full length.
(PDF)

S20 Fig Age positively correlates with several HCoV-specific antibody responses in healthy pre-pandemic children. The matrix depicts pairwise Spearman correlations between age and HCoV-specific antibody levels in healthy pre-pandemic samples. Only correlations with p < 0.1 after Benjamini-Hochberg adjustment for multiple comparisons are shown. Color and coefficient reflect the direction and magnitude of each pairwise correlation.
(PDF)

S21 Fig Clustering of serology does not separate by cancer status or tumor type. Hierarchical clustering of z-scored SARS-CoV-2 specific **(a, c)**, and HCoV-specific **(b,d)** antibody features from participants sampled pre-pandemic (top row) or post-pandemic in 2022 (bottom row) with and without cancer.
(PDF)

S22 Fig Sensitivity of exposure classification schema to alternative variant-level voting thresholds. Samples were classified using modified rules requiring at least *X* of 7 variant-level RBD responses above pre-pandemic thresholds (*X* = 1-4; *X* = 4 is equivalent to the original majority voting schema) to classify prior exposure status. For each threshold, samples were assigned to recent, remote, cross-reactive, or non-reactive groups based on N and RBD seropositivity, shown as proportions in pie charts. Exposure assignments did not differ significantly between healthy children and children with cancer sampled in 2022 by Fisher’s exact test across all thresholds (1 variant: p = 0.64; 2 variants: p = 0.15; 3 variants: p = 0.79; 4 variants: p = 0.83).
(PDF)
