# Supplementary material for: Estimating SARS-CoV-2 exposure in asymptomatic hospitalized children with cancer in Western Kenya: A retrospective analysis of serological data
Source: PLoS One. 2026 Jul 10;21(7):e0353284. doi: 10.1371/journal.pone.0353284 (PMC13354098; doi:10.1371/journal.pone.0353284)
Supplement: S1 Appendix — (PDF) [file pone.0353284.s001.pdf]

S1 Appendix: Estimating SARS-CoV-2 exposure in asymptomatic hospitalized children with cancer in Western Kenya: a retrospective analysis of serological data

S1 Appendix. Luminex assay supplementary methods

Sample Collection and Preparation

Venous blood was collected in sodium heparin BD Vacutainers and processed within 2 hours. Plasma was separated by centrifugation at 1,000g for 10 minutes and stored at -20°C until use. On the day prior to the Luminex assay, plasma samples were thawed and centrifuged at 10,000g for 10 minutes to pellet any aggregates. Clarified plasma was then collected and diluted (1:100 and 1:500) using ABE buffer (PBS, 0.1% BSA, 0.05% Tween-20, and 0.05% sodium azide; pH 7.4).

Determination of Isotype/Subclass Levels and FcγR Binding of Anti–SARS-CoV-2 Antibodies by Luminex Assay

Luminex magnetic beads (MagPlex Microspheres) were coupled to the antigens listed in appendix p3, using a distinct bead region for each antigen. Coupling was performed according to the Luminex xMAP Cookbook (4th edition). Briefly, 5 million beads from each region were washed with distilled water and activated for 20 minutes on a rotator in the dark using EDC and Sulfo-NHS (50 mg/mL each) in activation buffer (0.1 M monosodium phosphate, pH 6.2). After three washes with MES buffer (0.05 M MES, pH 5.0), the appropriate amount of each protein (appendix p 4) was added, and beads were incubated for 2 hours on a rotator in the dark. Beads were then washed three times with storage buffer (PBS, 0.1% BSA, 0.02% Tween-20, and 0.05% sodium azide; pH 7.4), counted using a hemocytometer, and stored at 4°C in the dark until use. A day prior to the assay, a bead mix was prepared to allow for distribution of 50 µL per well (384-well plate), containing 500 beads per region per well. Following bead addition and washing, 50 µL of 4-fold serially diluted standards (a mixture of pre-pandemic plasma from healthy African adults, plasma from a vaccinated American adult, and plasma from a SARS-CoV-2-infected patient) and 50 µL of prepared samples were added to the appropriate wells. Sample dilutions in ABE buffer were as follows: 1:100 for IgG2, IgG3, IgG4, IgA1, and IgM; 1:500 for total IgG and IgG1; and 1:1000 for FcγR binding assessments. Plates were incubated on a shaker at 750 rpm for 2 hours at room temperature in the dark. After three washes with ABE buffer, 50 µL of either PE-conjugated secondary antibody or PE-conjugated FcγR (1:200) was added to each well, followed by incubation on a shaker at 750 rpm for 1 hour in the dark. The PE-conjugated antibodies included: mouse anti-human total IgG-PE (Southern Biotech, Cat#9040-09, 1:100), IgG1-PE (Cat#9052-09, 1:100), IgG2-PE (Cat#9070-09, 1:200), IgG3-PE (Cat#9210-09, 1:100), IgG4-PE (Cat#9200-09, 1:200), IgA1-PE (Cat#9130-09, 1:50), and IgA2-PE (Cat#9140-09, 1:100). Following three additional washes with ABE, 60 µL of ABE buffer was added per well. Plates were briefly shaken (30 seconds at 750 rpm) and read on a FlexMap3D (Luminex). The instrument acquired a minimum of 50 beads per bead region per well to determine the median fluorescence intensity (MFI). Linearity of standard curves was assessed to confirm assay validity prior to analysis.

Preparation of PE-FcγR Reagents

FcγR2A and FcγR3A proteins were obtained from the Duke Human Vaccine Institute Protein Production Facility. Biotinylation was performed using the BirA biotin-protein ligase standard reaction kit (Avidity, Cat#BirA500-BirA500) according to previously described and validated methods. Biotinylated FcγRs were snap-frozen in liquid nitrogen and stored at -80°C until use. One day before the assay, streptavidin-SAPE (Agilent, Cat#PJ315-1) was added to the biotinylated FcγRs to generate the PE-FcγR conjugates used in the Luminex assay.

S1 Appendix. k-NN cluster modeling supplementary methods

k-Nearest Neighbors (k-NN) Classification

We applied a k-nearest neighbors (k-NN) supervised classification algorithm to assign seroreactivity labels to samples from children with cancer based on their similarity to healthy children with known seroreactivity phenotypes. In this context, labeled samples refer to healthy children whose seroreactivity group (high *vs* low) had already been determined (via hierarchical clustering in Figure 2b), while unlabeled samples refer to children with cancer whose group membership was unknown and needed to be inferred.

To classify an new, unlabeled sample, k-NN computes its distance to all labeled samples in the training set, identifies the k nearest neighbors (those with the smallest distances in feature space), and assigns the most common class label among them. The value of k is a user-defined parameter; here, we used k=7 and Euclidean distance for all analyses.

Because k-NN relies on distance calculations, it can be sensitive to the scale and dimensionality of the data. In high-dimensional datasets, irrelevant or noisy features can distort distances and reduce classification accuracy. To address this, dimensionality reduction methods such as Principal Component Analysis (PCA) are often applied before k-NN. PCA transforms the original features into a smaller set of orthogonal components that capture the most variance in the data, helping to reduce noise and improve class separability.

Cross-Validation

To evaluate model performance within the training set (healthy samples) before applying it to cancer samples, we used *k*-fold cross-validation. In this approach, the dataset is split into *k* equally sized subsets (folds). The model is trained on *k*-1 folds and tested on the remaining fold, repeating this process *k* times so that each fold serves once as the test set. Accuracy, sensitivity, and specificity scores are averaged across folds to estimate the model's generalizability. Cross-validation also helps select the optimal number of neighbors (k) and assess robustness. Note: The value of *k* in cross-validation is not the same as k in k-NN.

Permutation-Based Significance Testing

To determine whether the model's accuracy was greater than expected by chance, we compared it to a null distribution generated by randomly permuting the sample labels, from samples in the training set, 1000 times. For each permutation, we repeated the classification procedure to produce a distribution of accuracies under the null hypothesis (i.e., no true relationship between features and labels). Statistical significance was assessed by calculating the proportion of permuted accuracies equal to or greater than the observed accuracy. This tests whether the model's performance was meaningfully better than random guessing.
