# Supplementary material for: Estimating SARS-CoV-2 exposure in asymptomatic hospitalized children with cancer in Western Kenya: A retrospective analysis of serological data
Source: PLoS One. 2026 Jul 10;21(7):e0353284. doi: 10.1371/journal.pone.0353284 (PMC13354098; doi:10.1371/journal.pone.0353284)
Supplement: S1 Table — (PDF) [file pone.0353284.s003.pdf]

S1 Table. Antigens in Luminex assay

Protein Name	Origin	Source	Cat #	Quantity used for coupling
RBD alpha	SARS-CoV-2	UMass Biologics	Gift from L. Cavacini	3.2 µg/ million beads
RBD beta	SARS-CoV-2	UMass Biologics	Gift from L. Cavacini	3.2 µg/ million beads
RBD delta	SARS-CoV-2	UMass Biologics	Gift from L. Cavacini	3.2 µg/ million beads
RBD gamma	SARS-CoV-2	UMass Biologics	Gift from L. Cavacini	3.2 µg/ million beads
RBD lambda	SARS-CoV-2	UMass Biologics	Gift from L. Cavacini	3.2 µg/ million beads
RBD omicron	SARS-CoV-2	UMass Biologics	Gift from L. Cavacini	3.2 µg/ million beads
FL Spike (S-trimer)	SARS-CoV-2	UMass Biologics	Gift from L. Cavacini	5.11 µg/ million beads
N (Nucleocapsid)	SARS-CoV-2	UMass Biologics	Gift from L. Cavacini	5.8 µg/ million beads
HCoV-NL63	Common Coronavirus	Sino Biology	40604-V08B	1.424 µg / million beads
HCoV-OC43	Common Coronavirus	Sino Biology	40607-V08B	1.451 µg / million beads
HCoV-HKU1	Common Coronavirus	Sino Biology	40021-V08H	0.858 µg / million beads
HCoV-229E	Common Coronavirus	Sino Biology	40605-V08B	1.22 µg / million beads
BSA	Internal Control	Sigma	A7030-500g	6.65 µg / million beads
