# Supplementary material for: Estimating SARS-CoV-2 exposure in asymptomatic hospitalized children with cancer in Western Kenya: A retrospective analysis of serological data
Source: PLoS One. 2026 Jul 10;21(7):e0353284. doi: 10.1371/journal.pone.0353284 (PMC13354098; doi:10.1371/journal.pone.0353284)
Supplement: S2 Table — (PDF) [file pone.0353284.s004.pdf]

S2 Table. Antibodies and FcRs in Luminex assay

Isotype/ Subclass	Antibody	Source	Cat #	Sample Dilution Factor†
Total IgG	Mouse Anti-Human IgG-PE	Southern Biotech	9040-09	1:100
IgG1	Mouse Anti-Human IgG1-PE	Southern Biotech	9052-09	1:100
IgG2	Mouse Anti-Human IgG2-PE	Southern Biotech	9070-09	1:200
IgG3	Mouse Anti-Human IgG3-PE	Southern Biotech	9210-09	1:100
IgG4	Mouse Anti-Human IgG4-PE	Southern Biotech	9200-09	1:200
IgA1	Mouse Anti-Human IgA1-PE	Southern Biotech	9130-09	1:50
IgA2	Mouse Anti-Human IgA2-PE	Southern Biotech	9140-09	1:100
FcγR2A	..	Duke Human Vaccine Institute Protein Production Facility	..	1:200
FcγR3A	..	Duke Human Vaccine Institute Protein Production Facility	..	1:200

†Dilution conducted with ABE buffer (PBS, 0.1% BSA, 0.05% Tween-20 and 0.05% sodium azide, pH=7.4)
