# Supplementary material for: Estimating SARS-CoV-2 exposure in asymptomatic hospitalized children with cancer in Western Kenya: A retrospective analysis of serological data
Source: PLoS One. 2026 Jul 10;21(7):e0353284. doi: 10.1371/journal.pone.0353284 (PMC13354098; doi:10.1371/journal.pone.0353284)
Supplement: S3 Table — (PDF) [file pone.0353284.s005.pdf]

S3 Table. Demographics of healthy participants by seroreactivity phenotype

	**Pre-Pandemic**		**Post-Pandemic**	
	Low Reactivity (n = 162)	High Reactivity (n = 20)	Low Reactivity (n = 89)	High Reactivity (n = 200)
**Site**‡ (No. (%))				
Chulaimbo	116 (72%)	17 (85%)	··	··
Mosoriot	46 (28%)	3 (15%)	21 (24%)	76 (38%)
Ahero	··	··	68 (76%)	124 (62%)
**Age**† (Mean (SD))	7.8 (2.4)	9.0 (3.4)	4.5 (1.0)	4.5 (1.1)
**Sex** = Male (%)	98 (60%)	17 (85%)	45 (51%)	94 (47%)
**Collection Year** (No. (%))				
2019	116 (72%)	17 (85%)	··	··
2020	46 (28%)	3 (15%)	··	··
2022	··	··	89 (100%)	200 (100%)

Mann-Whitney U or Fisher's exact test were used to determine significant differences
† Significant differences between *pre-pandemic* samples from low and high reactivity clusters
‡ Significant differences between *post-pandemic* samples from low and high reactivity clusters
