# Supplementary material for: Estimating SARS-CoV-2 exposure in asymptomatic hospitalized children with cancer in Western Kenya: A retrospective analysis of serological data
Source: PLoS One. 2026 Jul 10;21(7):e0353284. doi: 10.1371/journal.pone.0353284 (PMC13354098; doi:10.1371/journal.pone.0353284)
Supplement: S4 Table — (PDF) [file pone.0353284.s006.pdf]

S4 Table. Demographics of healthy post-pandemic participants by estimated exposure groups

	**Recent Infection** (n = 102)	**Remote Infection** (n = 46)	**Cross-reactive** (n = 26)	**Non-reactive** (n = 115)	P-value†
**Site** (No. (%))					
Mosoriot	50 (49%)	16 (35%)	7 (27%)	24 (21%)	<0.001
Ahero	52 (51%)	30 (65%)	19 (73%)	91 (79%)	
**Age** (Mean (SD))	4.6 (1.3)	4.3 (1.0)	4.4 (1.0)	4.6 (1.0)	0.49
**Sex** = Male (%)	49 (48%)	21 (46%)	12 (46%)	57 (50%)	0.97
**Seroreactivity Cluster** (No.)					
Low reactivity	0	2	4	83	..
High reactivity	102	44	22	32	

†Kruskal-Wallis or Fisher's exact test were used to determine significant differences
