# Supplementary material for: Estimating SARS-CoV-2 exposure in asymptomatic hospitalized children with cancer in Western Kenya: A retrospective analysis of serological data
Source: PLoS One. 2026 Jul 10;21(7):e0353284. doi: 10.1371/journal.pone.0353284 (PMC13354098; doi:10.1371/journal.pone.0353284)
Supplement: S5 Table — (PDF) [file pone.0353284.s007.pdf]

S5 Table. Demographics of post-pandemic cancer patients by seroreactivity phenotype

	**Low Reactivity** (n = 41)	**High Reactivity** (n = 32)	P-value†
**Site** (No. (%))			
MTRH	29 (71%)	30 (94%)	0.03
JOORTH	12 (29%)	2 (6%)	
**Age** (Mean (SD))	6.4 (3.8)	7.8 (3.6)	0.13
**Sex** = Male (%)	25 (61%)	19 (59%)	>0.99
**Collection Year** (No. (%))			
2020	20 (49%)	1 (3%)	<0.001
2021	11 (27%)	11 (34%)	
2022	10 (24%)	20 (63%)	

†Mann-Whitney U or Fisher's exact test were used to determine significant differences
