# Supplementary material for: Estimating SARS-CoV-2 exposure in asymptomatic hospitalized children with cancer in Western Kenya: A retrospective analysis of serological data
Source: PLoS One. 2026 Jul 10;21(7):e0353284. doi: 10.1371/journal.pone.0353284 (PMC13354098; doi:10.1371/journal.pone.0353284)
Supplement: S6 Table — (PDF) [file pone.0353284.s008.pdf]

S6 Table. Demographics of post-pandemic cancer patients by estimated exposure groups

	**Recent Infection** (n = 16)	**Remote Infection** (n = 7)	**Cross-reactive** (n = 3)	**Non-reactive** (n = 47)	P-value†
**Site** (No. (%))					
MTRH	15 (94%)	7 (100%)	2 (67%)	35 (75%)	0.17
JOORTH	1 (6%)	0 (0%)	1 (33%)	12 (25%)	
**Age** (Mean (SD))	7.3 (3.7)	10.3 (3.1)	3.2 (1.2)	6.7 (3.7)	0.03
**Sex** = Male (%)	7 (44%)	5 (71%)	3 (100%)	29 (62%)	0.24
**Seroreactivity Cluster** (No.)					
Low reactivity	0	0	1	40	..
High reactivity	16	7	2	7	
**Collection Year** (No. (%))					
2020	..	..	..	21 (45%)	<0.001
2021	3 (19%)	3 (43%)	..	16 (34%)	
2022	13 (81%)	4 (57%)	3 (100%)	10 (21%)	

†Kruskal-Wallis or Fisher's exact test were used to determine significant differences
