# Supplementary material for: Estimating SARS-CoV-2 exposure in asymptomatic hospitalized children with cancer in Western Kenya: A retrospective analysis of serological data
Source: PLoS One. 2026 Jul 10;21(7):e0353284. doi: 10.1371/journal.pone.0353284 (PMC13354098; doi:10.1371/journal.pone.0353284)
Supplement: S7 Table — (PDF) [file pone.0353284.s009.pdf]

S7 Table. Demographics of cancer patients sampled in 2022 by seroreactivity phenotype

	**Low Reactivity** (n = 10)	**High Reactivity** (n = 20)	P-value†
**Site** (No. (%))			
MTRH	7 (70%)	19 (95%)	0.18
JOORTH	3 (30%)	1 (5%)	
**Age** (Mean (SD))	5.2 (2.3)	7.1 (3.6)	0.13
**Sex** = Male (%)	7 (70%)	12 (60%)	0.89

†Mann-Whitney U or Fisher's exact test were used to determine significant differences
