# Supplementary material for: Estimating SARS-CoV-2 exposure in asymptomatic hospitalized children with cancer in Western Kenya: A retrospective analysis of serological data
Source: PLoS One. 2026 Jul 10;21(7):e0353284. doi: 10.1371/journal.pone.0353284 (PMC13354098; doi:10.1371/journal.pone.0353284)
Supplement: S8 Table — (PDF) [file pone.0353284.s010.pdf]

S8 Table. Demographics of cancer patients sampled in 2022 by estimated exposure groups

	**Recent Infection** (n = 13)	**Remote Infection** (n = 4)	**Cross-reactive** (n = 3)	**Non-reactive** (n = 10)	P-value†
**Site** (No. (%))					
MTRH	12 (92%)	4 (100%)	2 (67%)	8 (80%)	0.49
JOORTH	1 (8%)	0 (0%)	1 (33%)	2 (20%)	
**Age** (Mean (SD))	7.3 (3.9)	8.9 (2.6)	3.2 (1.2)	5.4 (2.1)	0.06
**Sex** = Male (%)	6 (46%)	3 (75%)	3 (100%)	7 (70%)	0.28
**Seroreactivity Cluster** (No.)					
Low reactivity	0	0	1	9	..
High reactivity	13	4	2	1	

†Kruskal-Wallis or Fisher's exact test were used to determine significant differences
