# Supplementary material for: Estimating SARS-CoV-2 exposure in asymptomatic hospitalized children with cancer in Western Kenya: A retrospective analysis of serological data
Source: PLoS One. 2026 Jul 10;21(7):e0353284. doi: 10.1371/journal.pone.0353284 (PMC13354098; doi:10.1371/journal.pone.0353284)
Supplement: S9 Table — (PDF) [file pone.0353284.s011.pdf]

S9 Table. Summary of cancer diagnoses in pre-pandemic participants

Cancer diagnosis	Number of Participants
Burkitt Lymphoma	12 (75%)
Rhabdomyosarcoma	2 (13%)
Hodgkins Lymphoma	1 (6%)
Nephroblastoma	1 (6%)
