# Supplementary material for: Estimating SARS-CoV-2 exposure in asymptomatic hospitalized children with cancer in Western Kenya: A retrospective analysis of serological data
Source: PLoS One. 2026 Jul 10;21(7):e0353284. doi: 10.1371/journal.pone.0353284 (PMC13354098; doi:10.1371/journal.pone.0353284)
Supplement: S10 Table — (PDF) [file pone.0353284.s012.pdf]

S10 Table. Summary of cancer diagnoses in post-pandemic participants

Cancer diagnosis	Number of Participants
Burkitt Lymphoma	52 (70%)
Hodgkins Lymphoma	4 (5.5%)
Nephroblastoma	3 (4.0%)
Neuroblastoma	2 (2.7%)
Non-Hodgkins Lymphoma	2 (2.7%)
Rhabdomyosarcoma	2 (2.7%)
Acute Lymphoblastic Leukemia	1 (1.3%)
Acute Lymphoma	1 (1.3%)
Hepatoblastoma	1 (1.3%)
Leukemia	1 (1.3%)
Myeloid Sarcoma	1 (1.3%)
Paraganglioma	1 (1.3%)
T- Lymphoblastic Lymphoma	1 (1.3%)
T-cell Lymphoma	1 (1.3%)
Yolk Sac Tumor	1 (1.3%)
