# Supplementary figures and images for: Estimating SARS-CoV-2 exposure in asymptomatic hospitalized children with cancer in Western Kenya: A retrospective analysis of serological data
Source: PLoS One. 2026 Jul 10;21(7):e0353284. doi: 10.1371/journal.pone.0353284 (PMC13354098; doi:10.1371/journal.pone.0353284)

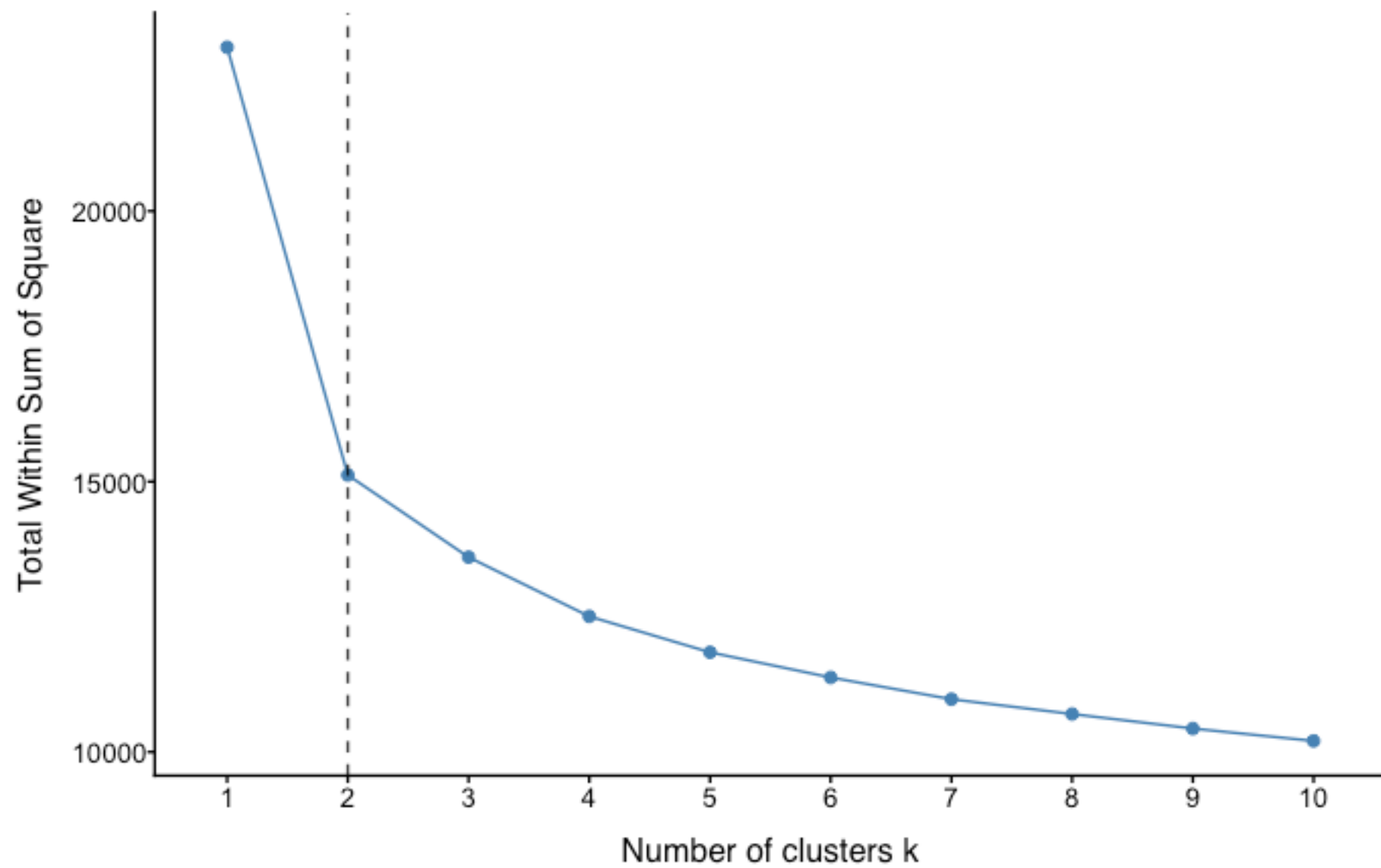

Supplement: S1 Fig — Elbow plot depicting the within-cluster sum of squares versus the number of clusters. The optimal cluster number for hierarchical clustering was determined by the inflection point, denoted by the dashed line. (PDF) [file pone.0353284.s013.pdf]

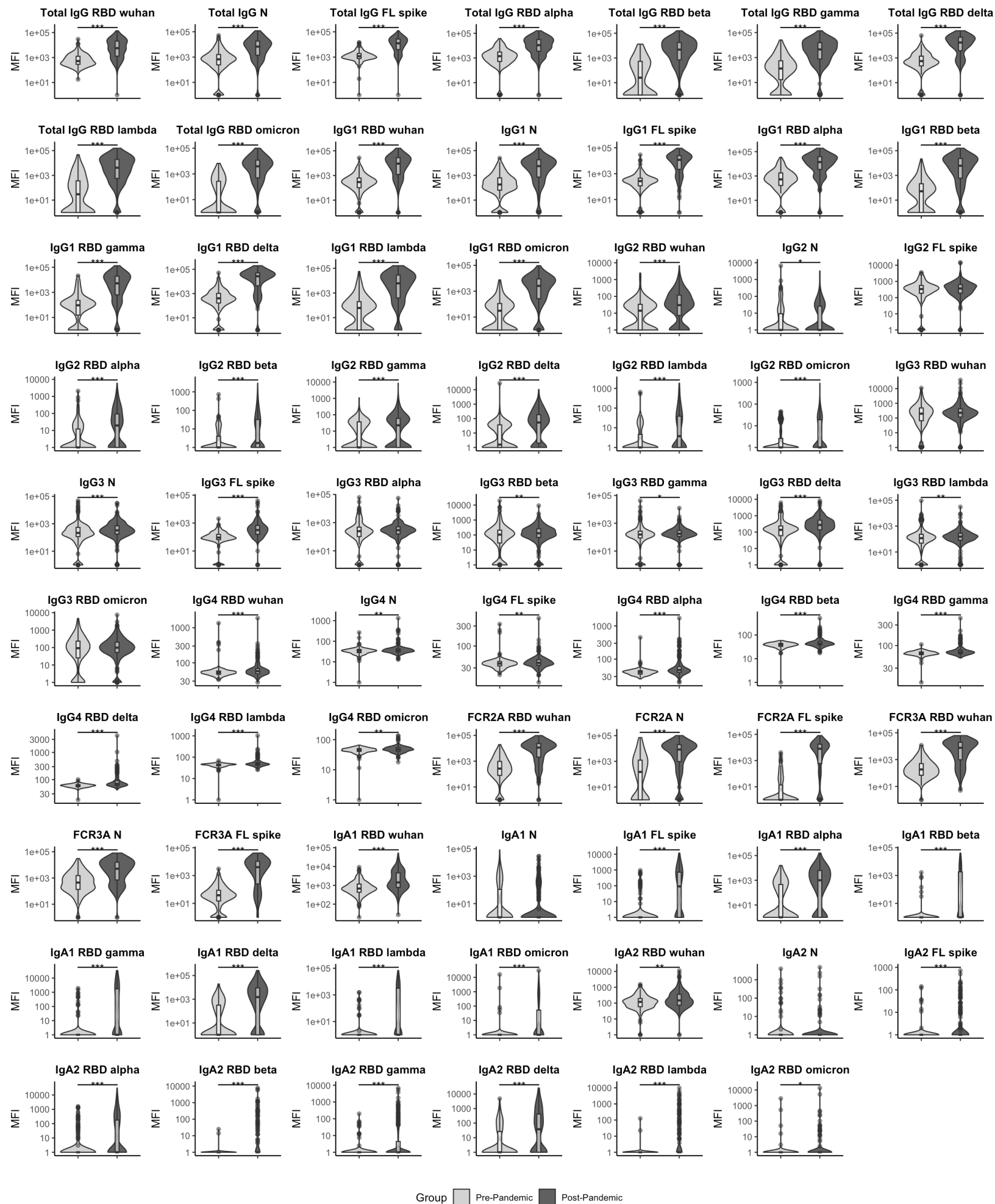

Supplement: S3 Fig — SARS-CoV-2-specific antibody levels in healthy children sampled pre-pandemic (light grey; n = 184) and post-pandemic (dark grey; n = 290). Statistical significance was assessed using the Mann-Whitney U test, with multiple comparisons adjusted by the Benjamini-Hochberg procedure (*p < 0.05, **p < 0.01, ***p < 0.001). N: nucleocapsid, RBD: receptor binding domain, MFI: mean fluorescence intensity. (PDF) [file pone.0353284.s015.pdf]

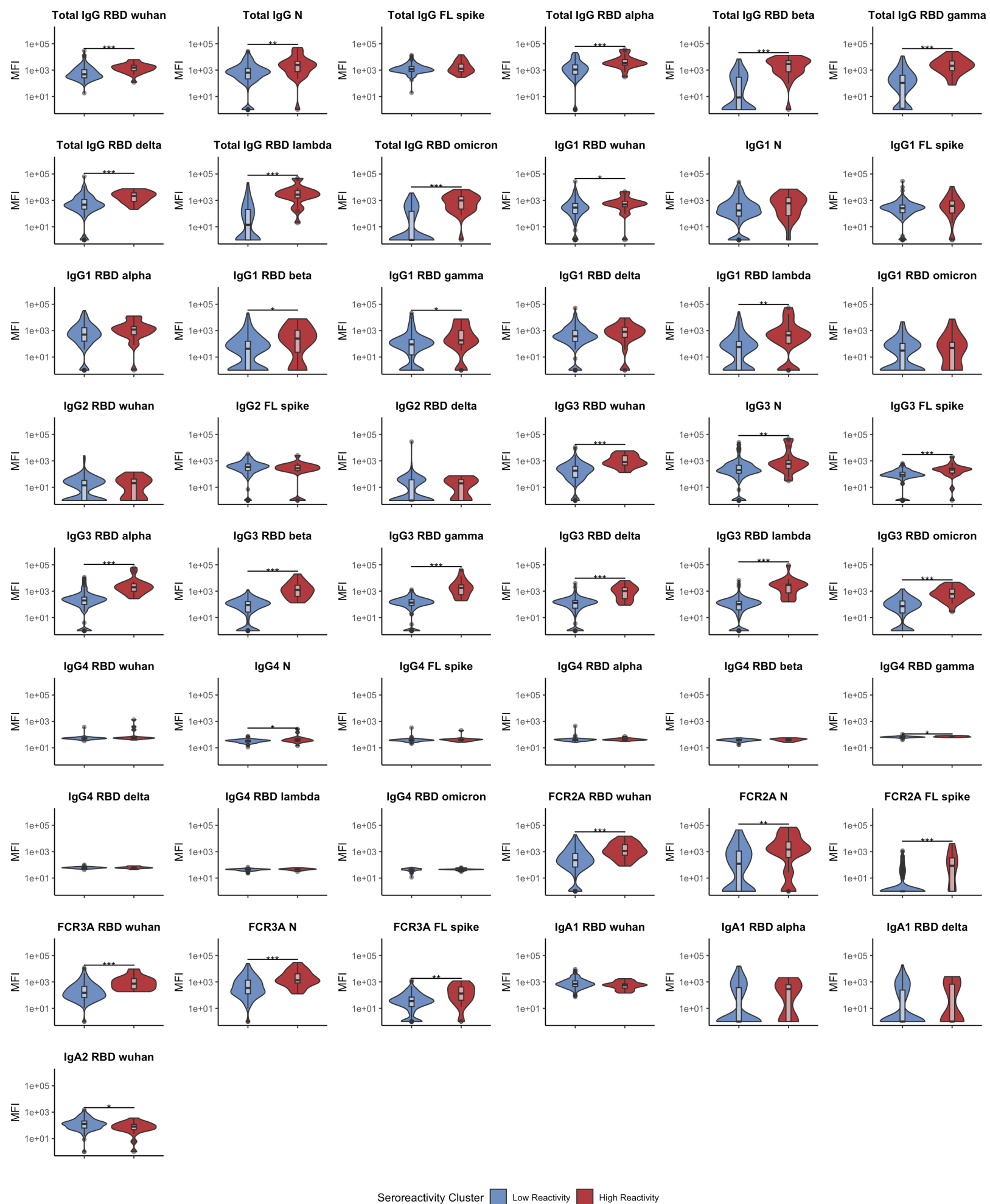

Supplement: S4 Fig — Comparisons of SARS-CoV-2-specific antibody levels in healthy children sampled pre-pandemic who clustered in the high (red; n = 20) and low (blue; n = 162) seroreactivity groups (as in Fig 2b). Statistical significance was assessed using the Mann-Whitney U test, with multiple comparisons adjusted by the Benjamini-Hochberg procedure (*p < 0.05, **p < 0.01, ***p < 0.001). N: nucleocapsid, RBD: receptor binding domain, MFI: mean fluorescence intensity. (PDF) [file pone.0353284.s016.pdf]

## Low Reactivity

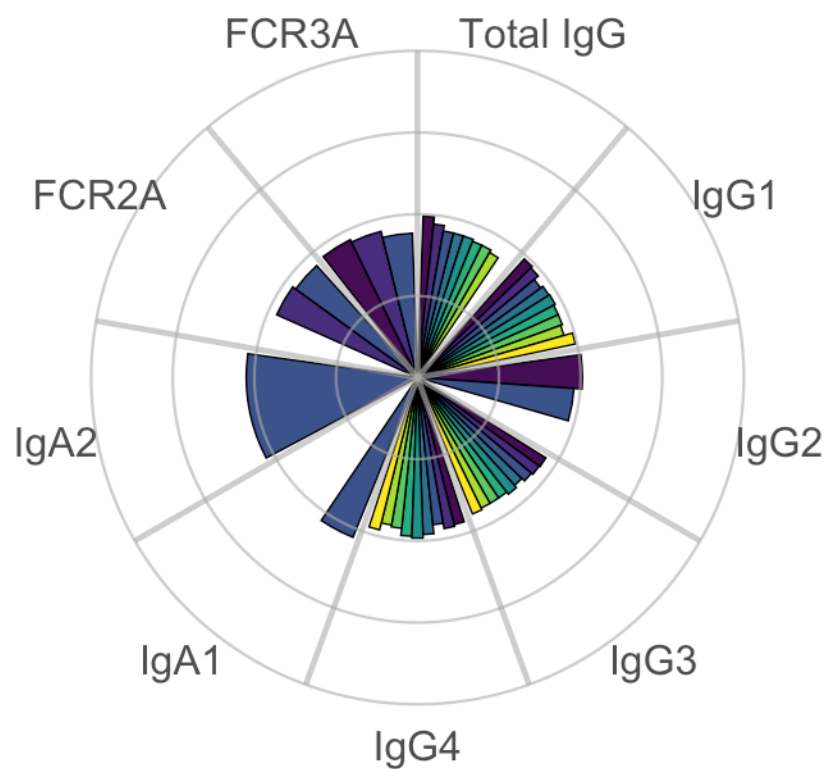

## High Reactivity

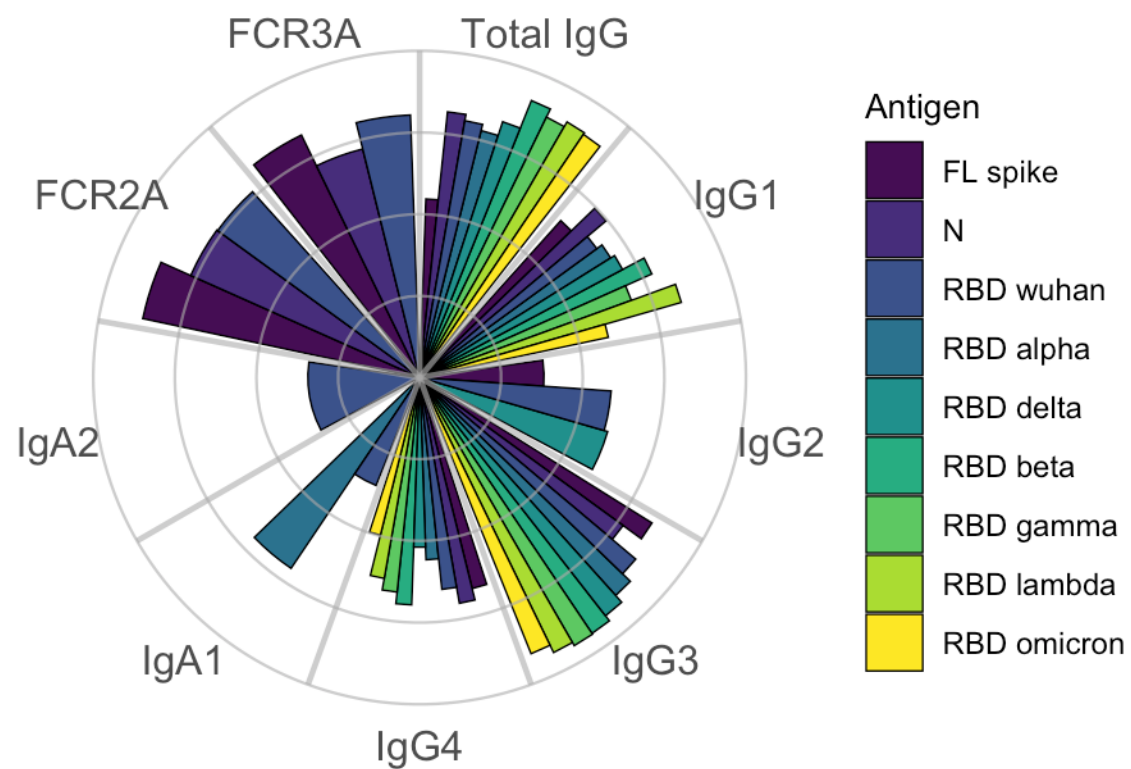

Supplement: S5 Fig — Polar plots depict the median percentile rank for each SARS-CoV-2 antibody feature from plasma samples from pre-pandemic healthy children who clustered in the low (left; n = 162) and high (right; n = 20) seroreactivity clusters (as in Fig 2b). Each wedge represents an antibody feature, and the size of the wedge depicts the median percentile ranging from 0 to 1. (PDF) [file pone.0353284.s017.pdf]

a -- 95<sup>th</sup> %tile of pre-pandemic samples

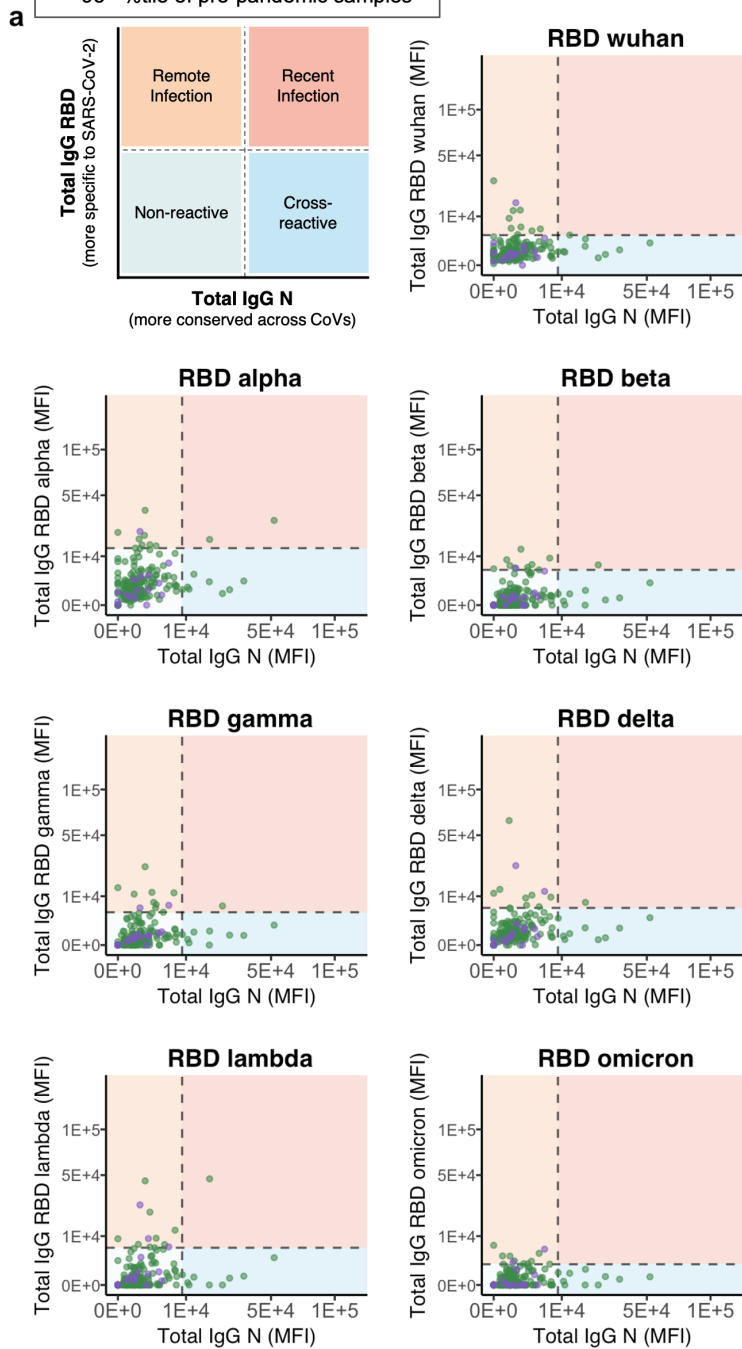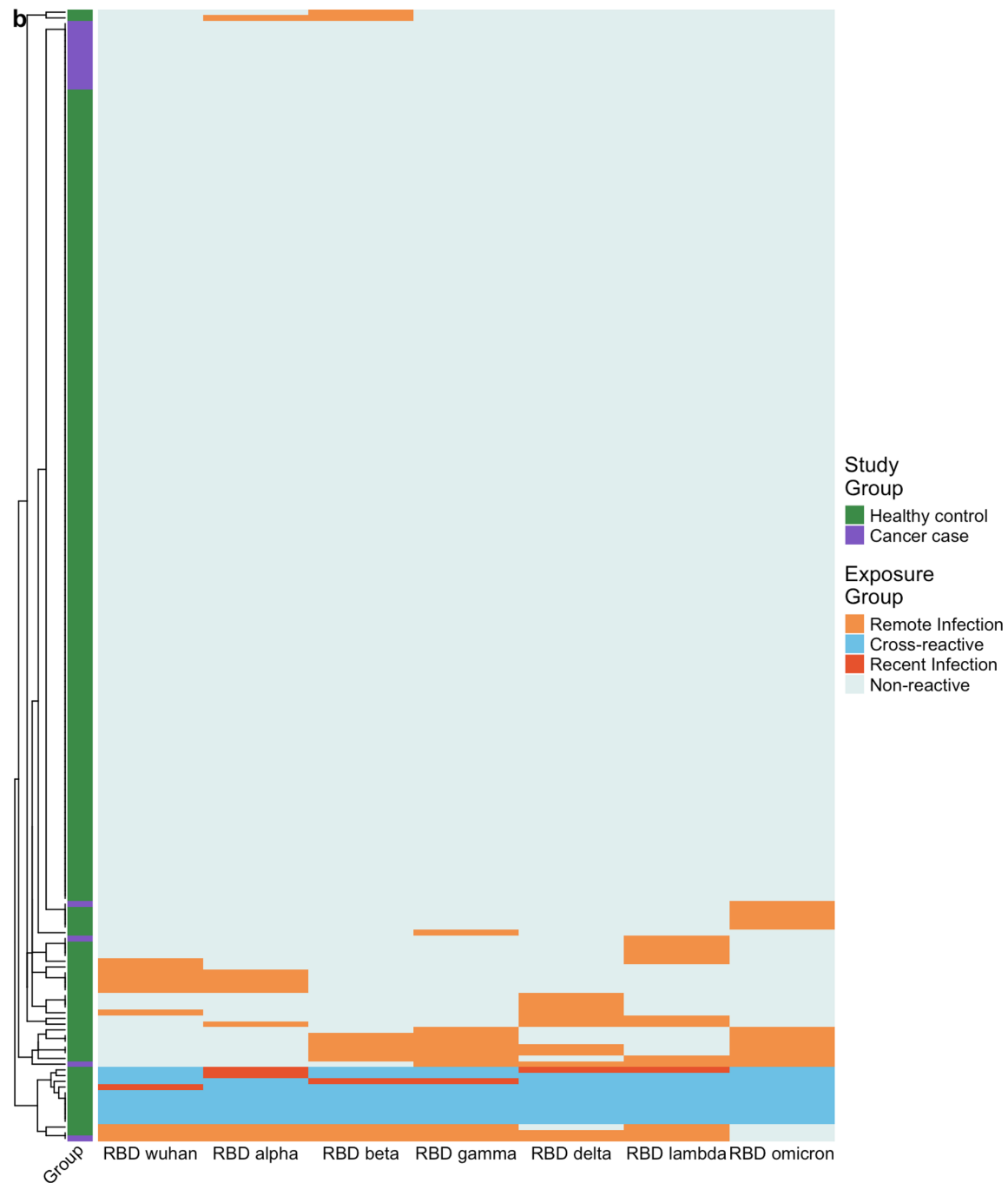

Supplement: S6 Fig — (a) Scatter plots of anti-N and anti-RBD antibody levels for seven RBD variants measured in pre-pandemic children with (purple) and without (green) cancer. Dashed lines represent the 95th percentile of healthy pre-pandemic samples, defining quadrants as shown in the upper-left cartoon. (b) Hierarchical clustering of exposure classifications for pre-pandemic samples across RBD variants. Final classifications for pre-pandemic samples were: 91% non-reactive (181/198), 5% cross-reactive (10/198), and 4% remote infection (7/198). N: nucleocapsid, RBD: receptor binding domain, MFI: mean fluorescence intensity. (PDF) [file pone.0353284.s018.pdf]

a

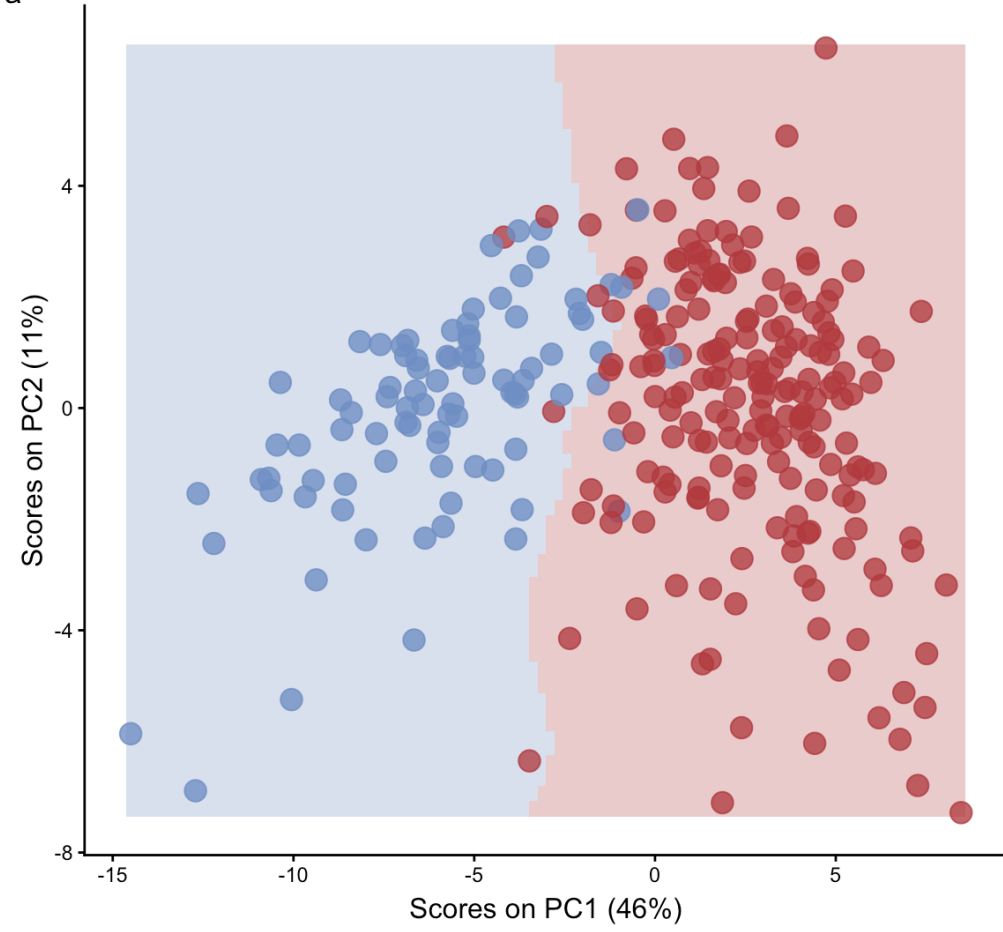

b

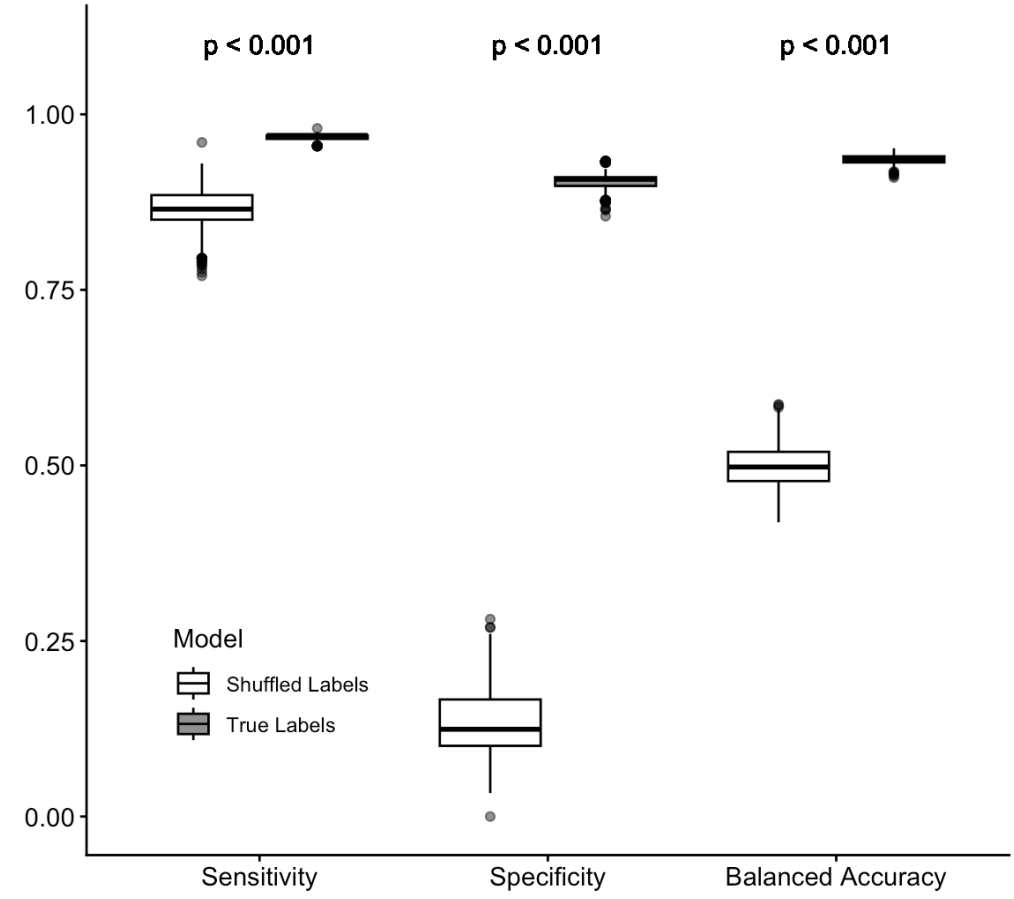

Supplement: S7 Fig — (a) Classification boundaries generated using k-nearest neighbors (k-NN) to distinguish samples with low (blue) and high (red) SARS-CoV-2 seroreactivity. The k-NN model was trained on the first three principal components of PCA-transformed serology data from healthy children sampled post-pandemic. Data points are colored based on hierarchical clustering classification (as in Fig 2b), while background shading indicates k-NN decision boundaries. (b) Performance of the k-NN model in classifying seroreactivity phenotypes among healthy post-pandemic samples when evaluated by 5-fold cross-validation. The model achieved 94% accuracy, 96% sensitivity, and 91% specificity. These values are shown relative to the cross-validated accuracy from 1000 label-permuted datasets. P-values were derived from the tail probability of the null distribution. PC: principal component. (PDF) [file pone.0353284.s019.pdf]

– 95<sup>th</sup> %tile of pre-pandemic samples

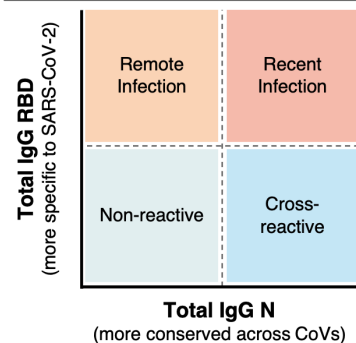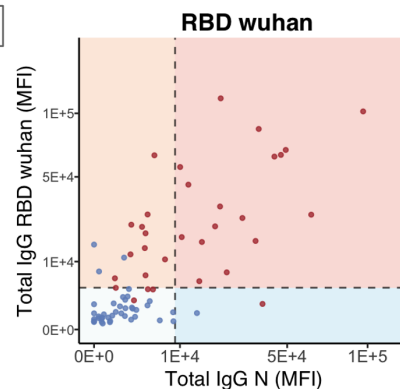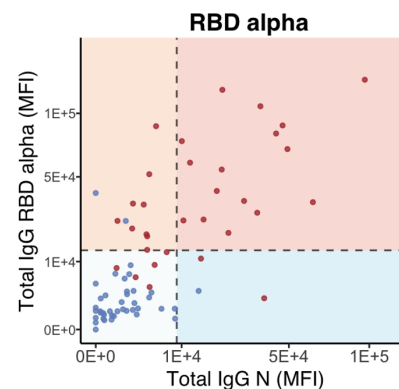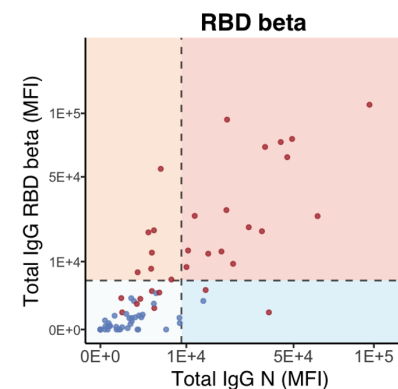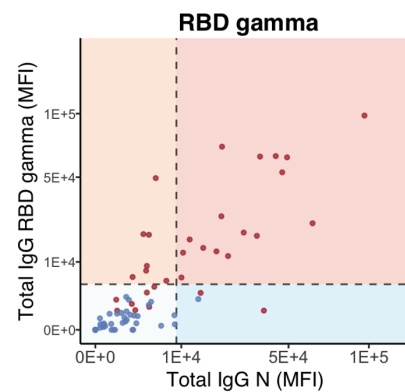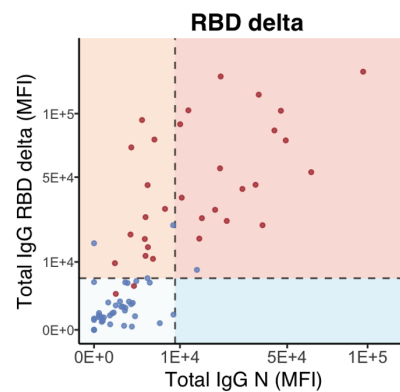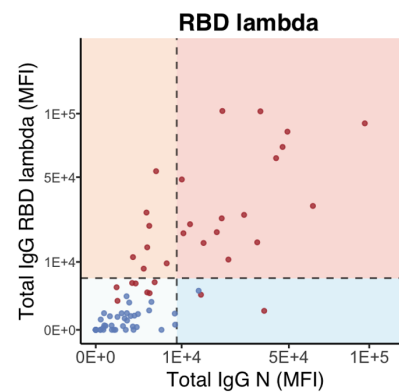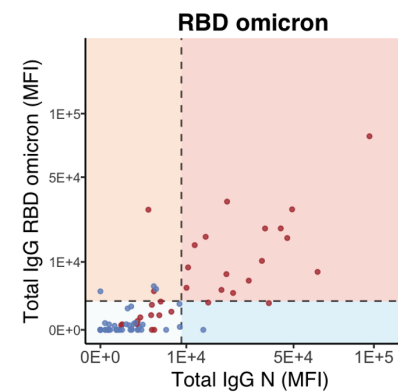

Supplement: S8 Fig — Scatter plots of Total IgG N and RBD antibody levels for seven RBD variants measured in post-pandemic children with cancer, colored by SARS-CoV-2 reactivity phenotypes (as in Fig 2b). Dashed lines represent the 95th percentile of healthy pre-pandemic samples, defining quadrants according to the color scheme in the upper left cartoon. Post-pandemic samples exceeding the 95th percentile of pre-pandemic controls for both N and RBD were classified as ‘recent infection’ (red). Samples exceeding the RBD threshold but not N were labeled as ‘remote infection’ (orange), while those exceeding the N threshold but not RBD were considered ‘cross-reactive’ (light blue). Samples below both thresholds were classified as ‘non-reactive’ (grey) for SARS-CoV-2 exposure. N: nucleocapsid, RBD: receptor binding domain, MFI: mean fluorescence intensity. (PDF) [file pone.0353284.s020.pdf]

a

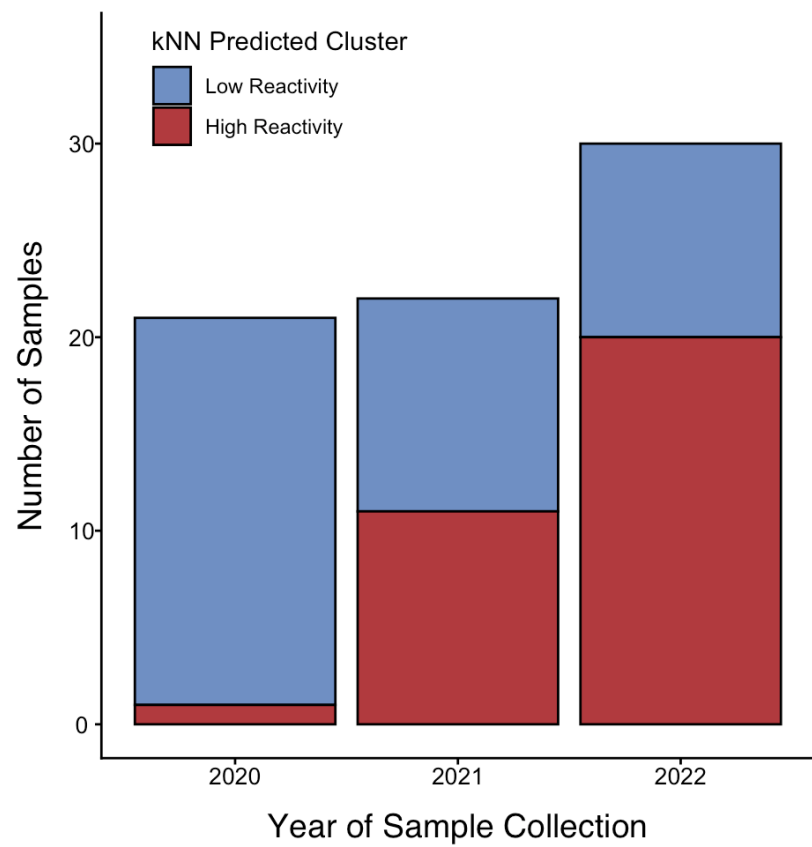

b

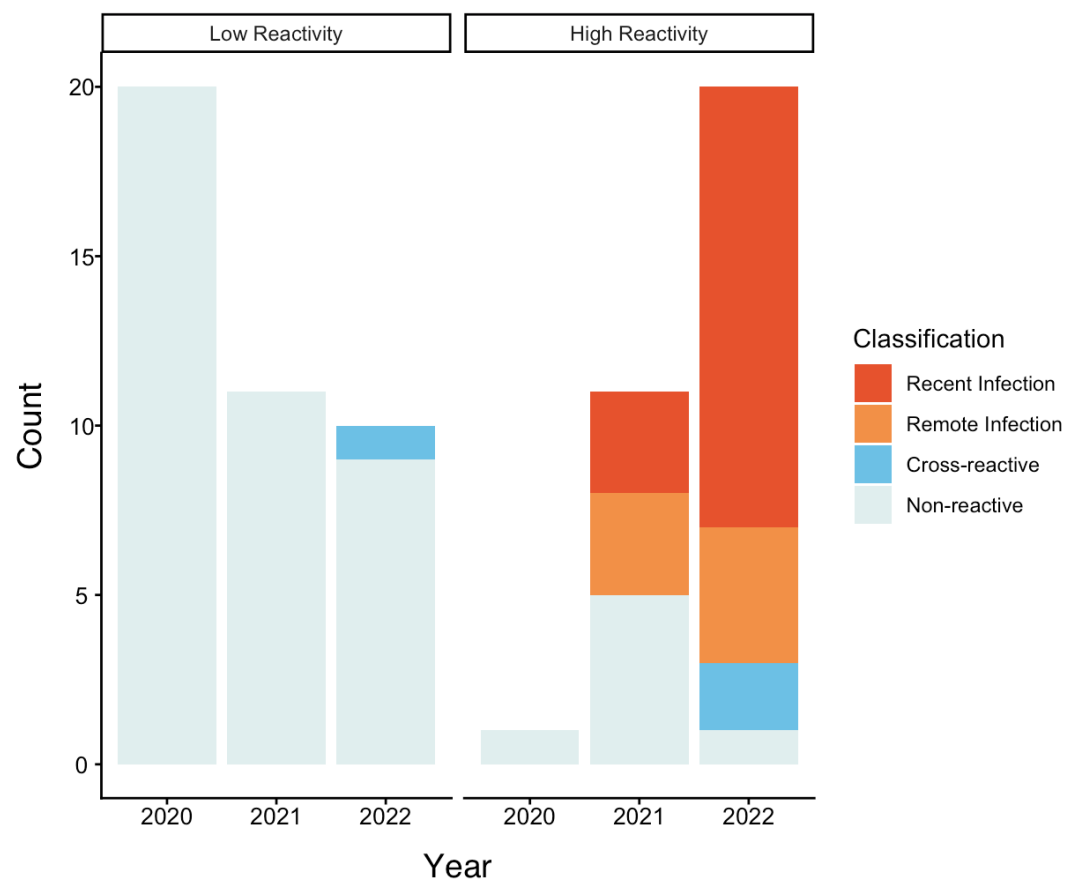

Supplement: S9 Fig — Stacked bar plots depicting the number of post-pandemic children with cancer classified into each seroreactivity phenotype (a) and SARS-CoV-2 exposure classification (b) by year of sample collection. kNN: k-nearest neighbor. (PDF) [file pone.0353284.s021.pdf]

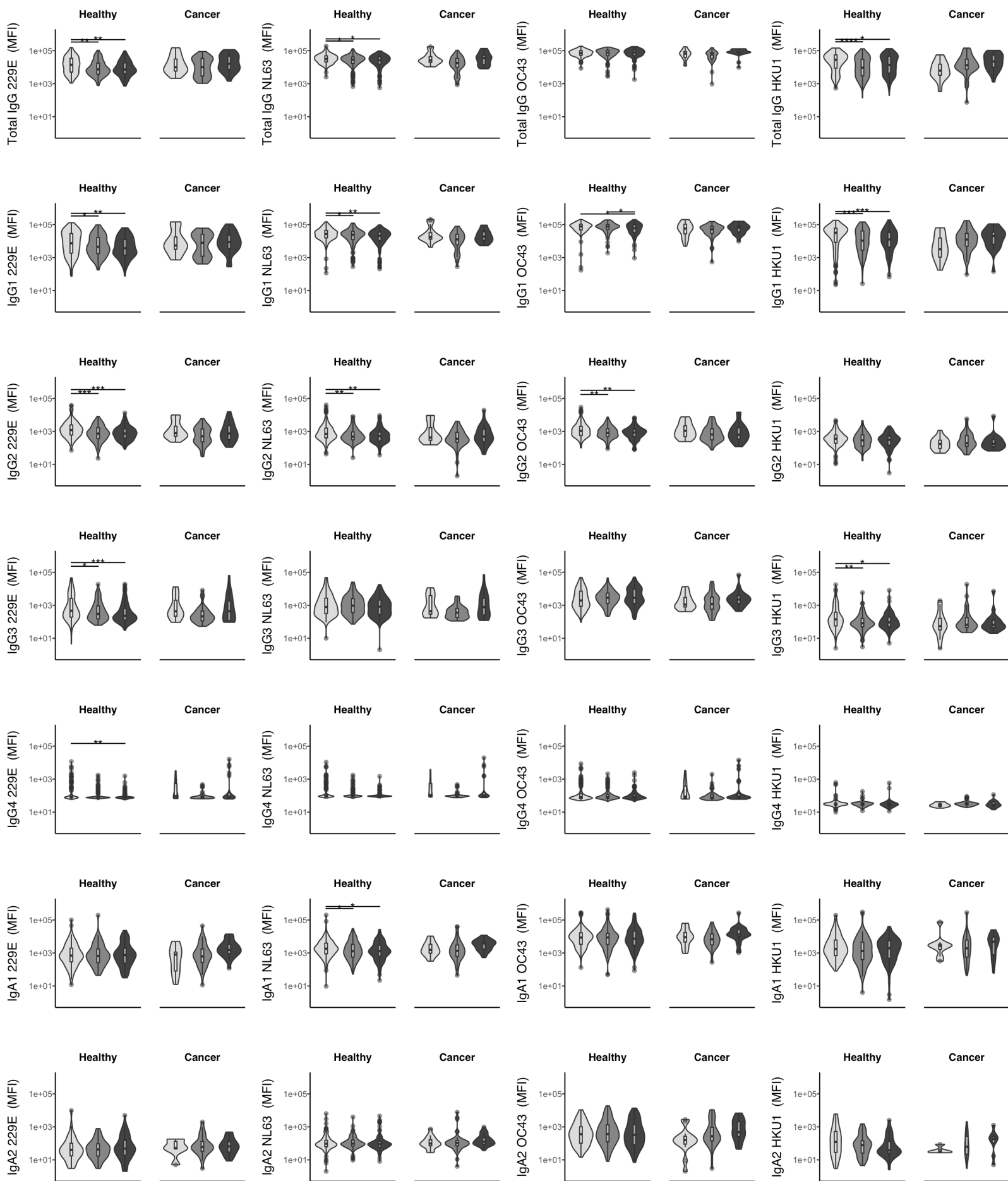

Pre-Pandemic Unexposed (Post-Pandemic) Exposed (Post-Pandemic)

Supplement: S10 Fig — HCoV-specific antibody levels in healthy children (left) and children with cancer (right), grouped by exposure type. Post-pandemic samples were categorized as exposed (recent or past infection) or unexposed (non-reactive or cross-reactive) based on final exposure classification (as in Fig 5). Statistical significance was assessed using the Kruskal-Wallis test with Benjamini-Hochberg correction for multiple comparisons. Dunn’s post hoc test was applied to significant results (*p < 0.05, **p < 0.01, ***p < 0.001, ****p < 0.0001). MFI: mean fluorescence intensity. (PDF) [file pone.0353284.s022.pdf]

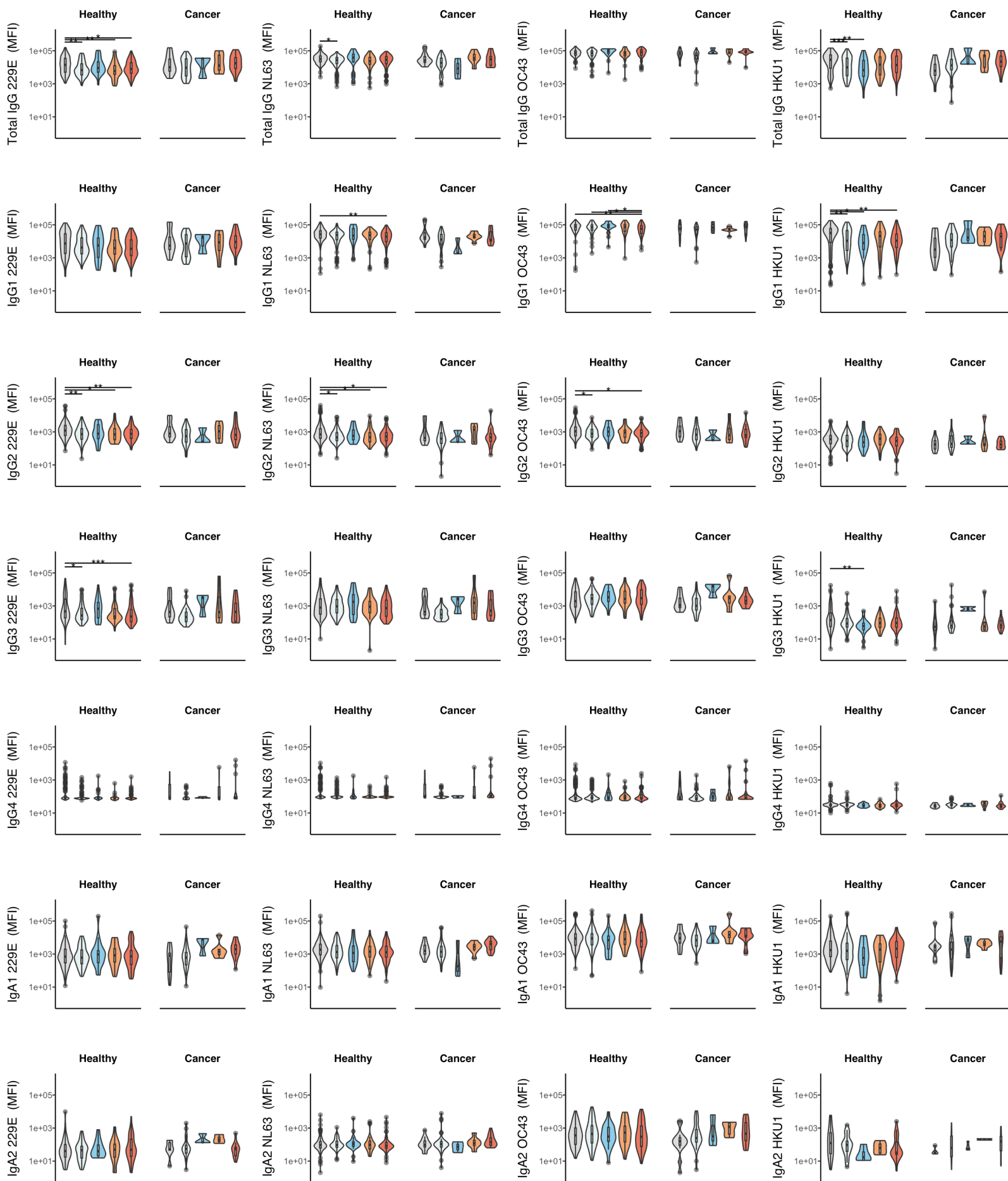

Pre-Pandemic Non-reactive Cross-reactive Remote Infection Recent Infection

Supplement: S11 Fig — HCoV specific antibody levels from healthy children (left) and children with cancer (right) based on final exposure classification. Statistical significance was assessed using the Kruskal-Wallis test with Benjamini-Hochberg correction for multiple comparisons. Dunn’s post hoc test was applied to significant results (*p < 0.05, **p < 0.01, ***p < 0.001, ****p < 0.0001). MFI: mean fluorescence intensity. (PDF) [file pone.0353284.s023.pdf]

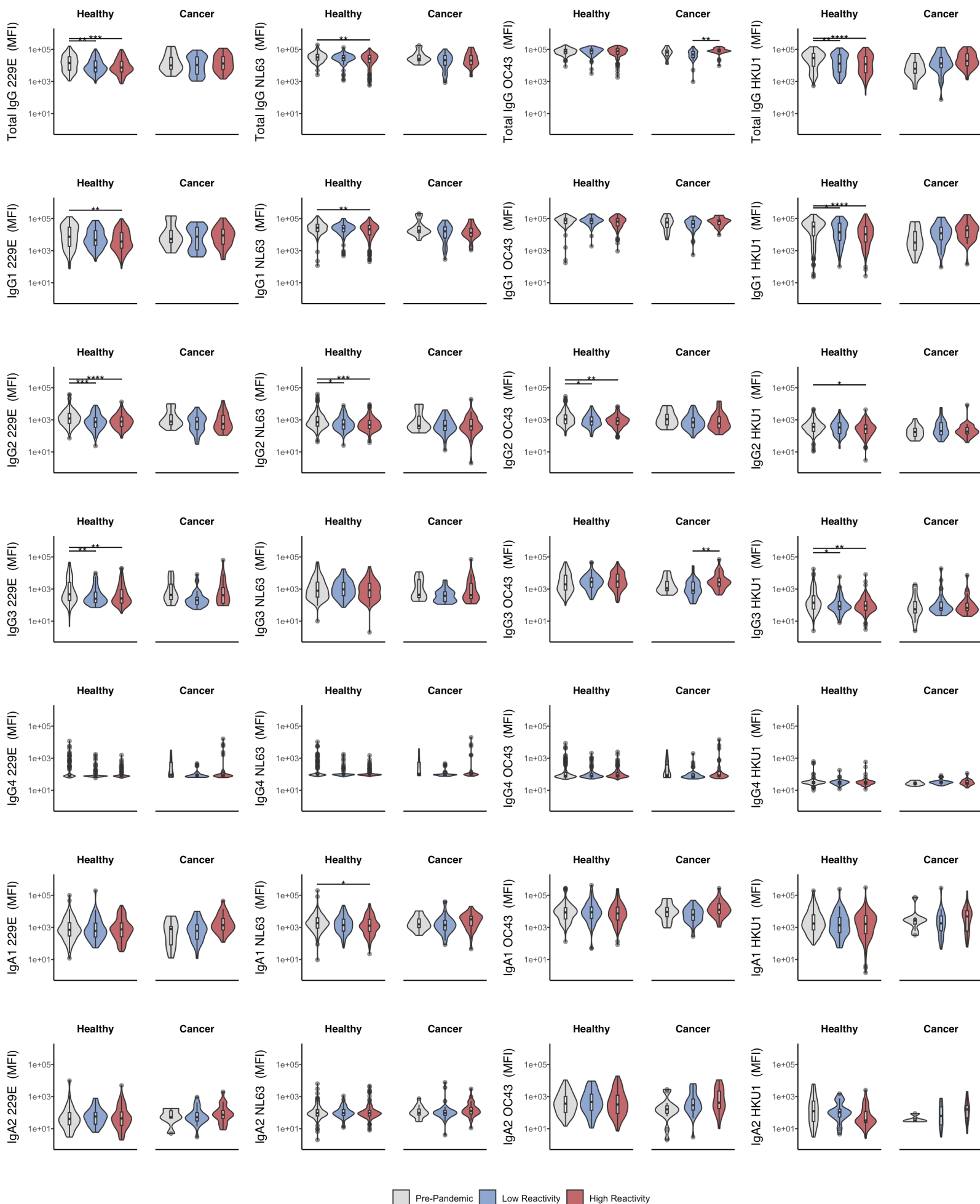

Supplement: S12 Fig — HCoV specific antibody levels from healthy children (left) and children with cancer (right) based on seroreactivity phenotype. Statistical significance was assessed using the Kruskal-Wallis test with Benjamini-Hochberg correction for multiple comparisons. Dunn’s post hoc test was applied to significant results (*p < 0.05, **p < 0.01, ***p < 0.001, ****p < 0.0001). MFI: mean fluorescence intensity. (PDF) [file pone.0353284.s024.pdf]

**a**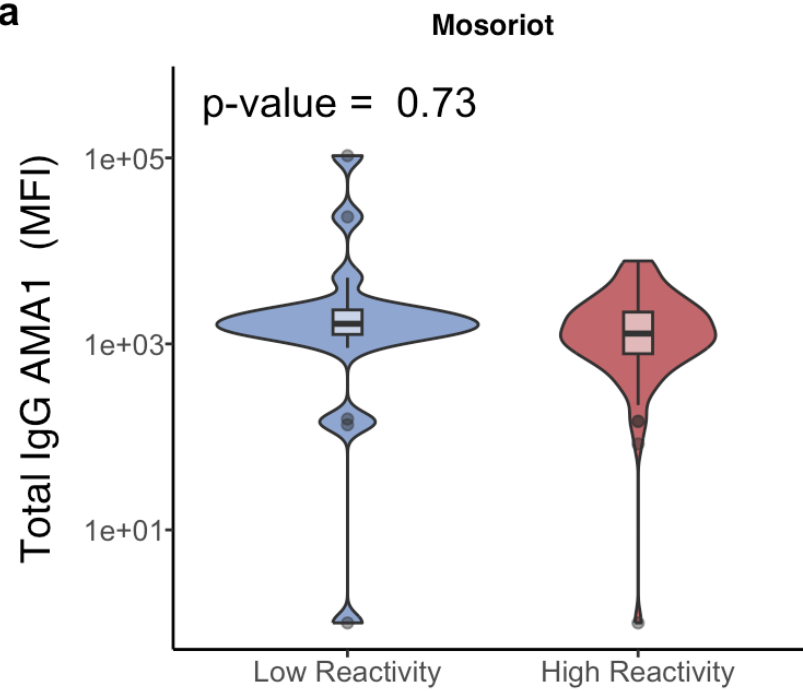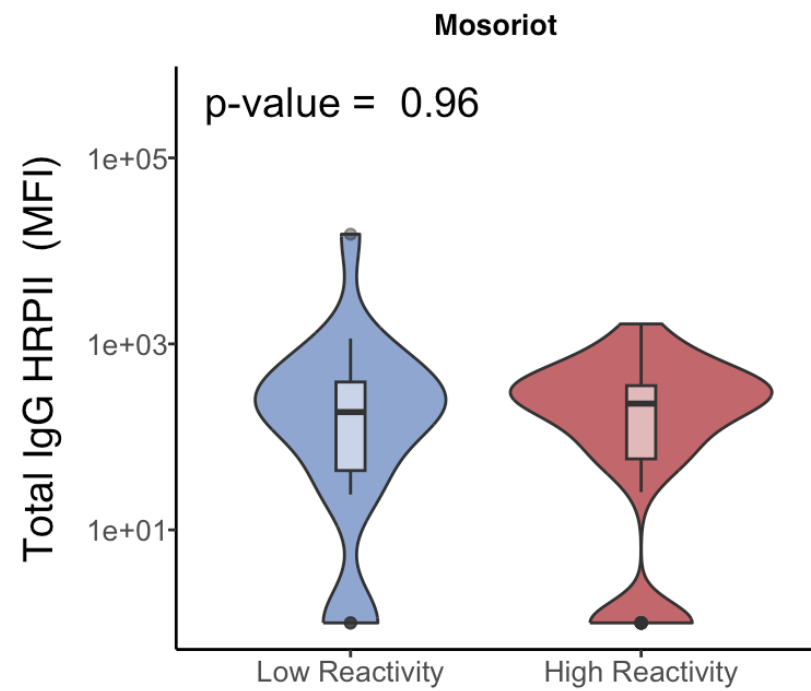**b**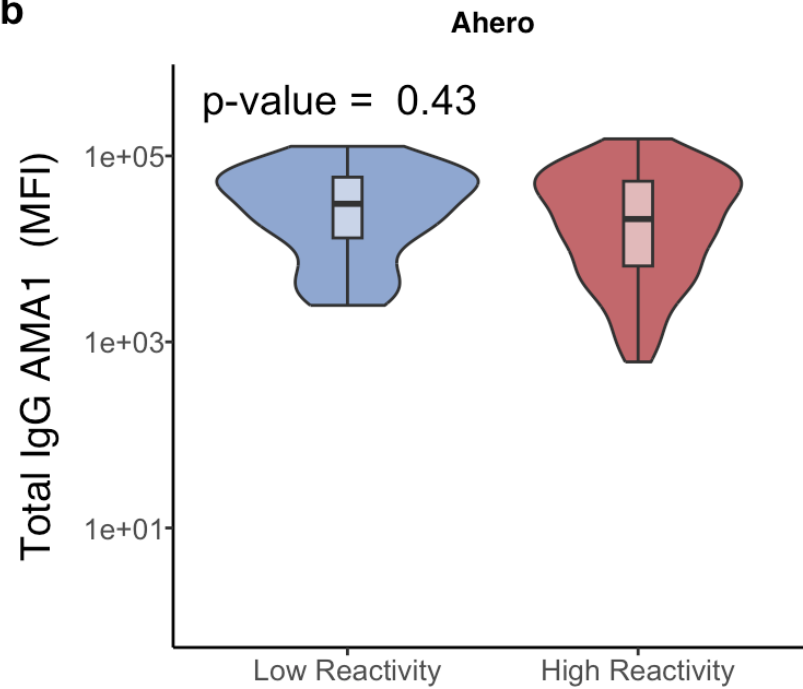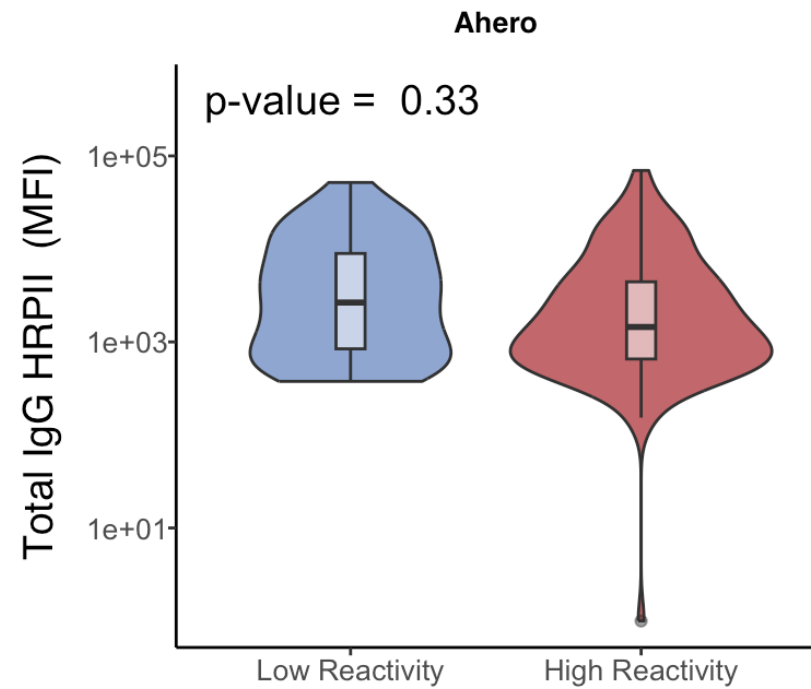

Supplement: S13 Fig — Comparison of Total IgG AMA1 and HRPII levels in healthy, post-pandemic children from Mosoriot (a) and Ahero (b) stratified by seroreactivity clusters (as in Fig 2b). AMA1 is a surrogate to indicate whether an individual was ever infected with malaria, and HRPII is a surrogate of recent infection. Statistical significance was assessed using the Mann-Whitney U test, with multiple comparisons adjusted by the Benjamini-Hochberg procedure. MFI: mean fluorescence intensity. (PDF) [file pone.0353284.s025.pdf]

**a** -- 95<sup>th</sup> %tile of pre-pandemic samples

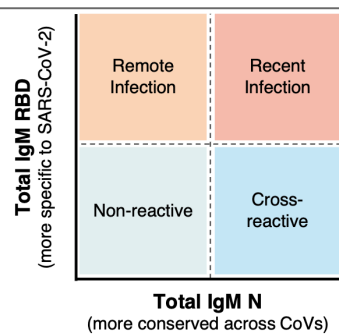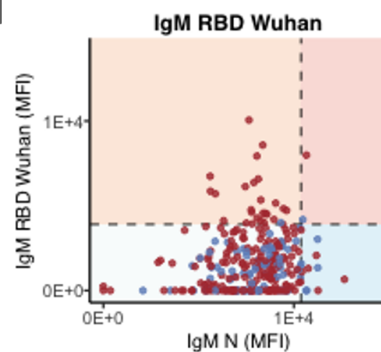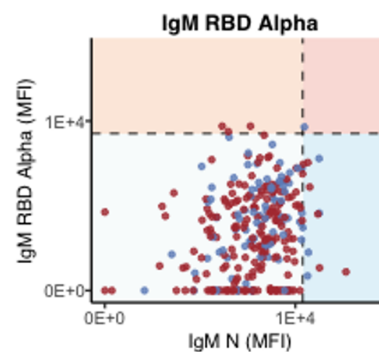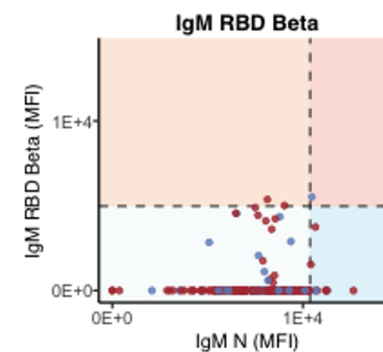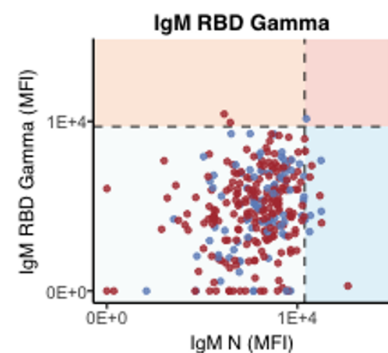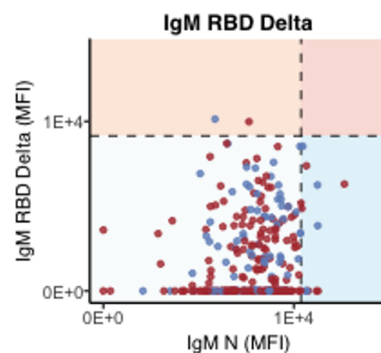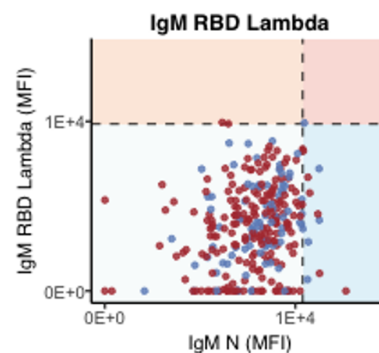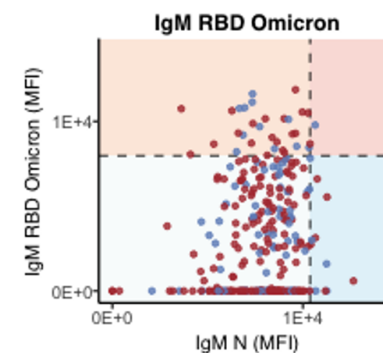

**b**

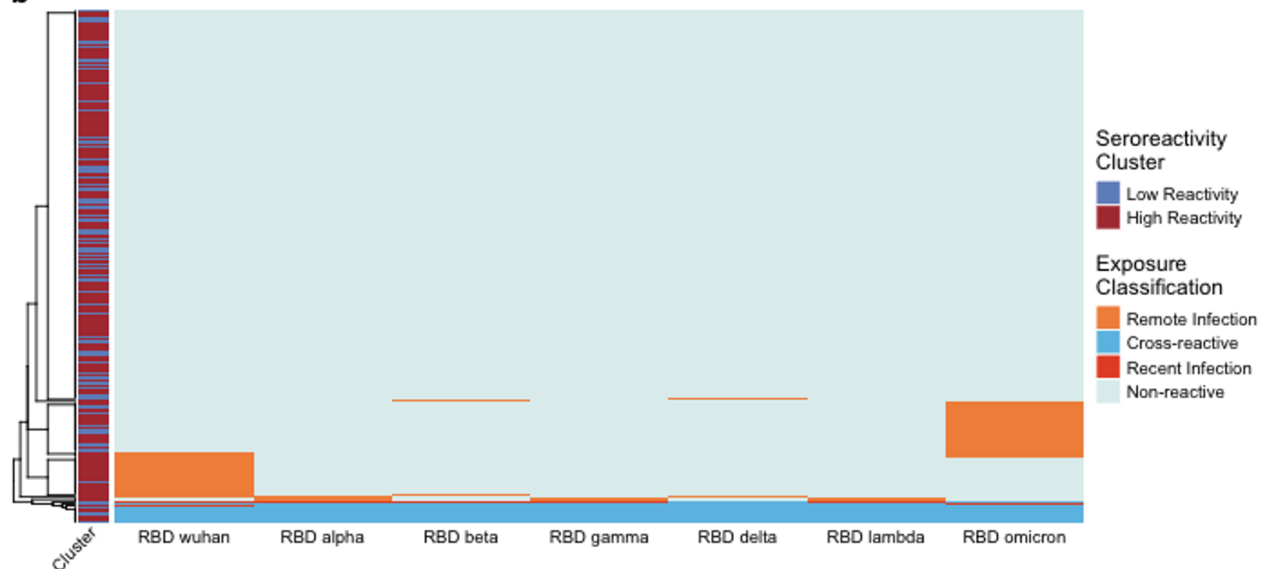

**c**

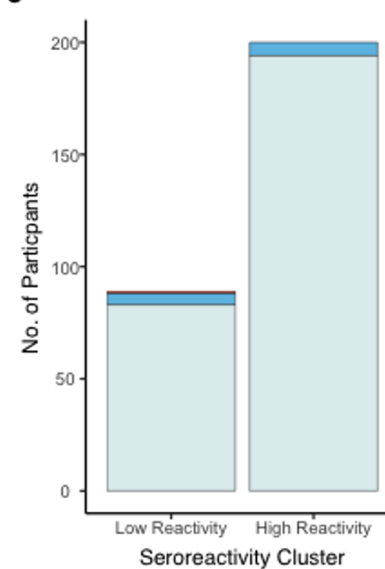

Supplement: S14 Fig — (a) Scatter plots of IgM N and RBD antibody levels for each RBD variant measured in post-pandemic healthy children from high (dark red) and low (dark blue) SARS-CoV-2 seroreactivity clusters (as in Fig 2b). Dashed lines represent the 95th percentile of healthy pre-pandemic samples, defining quadrants as shown in the upper-left cartoon. (b) Hierarchical clustering of exposure classifications for post-pandemic samples across RBD variants. (c) Stacked bar chart showing the number of samples classified in each exposure group, stratified by seroreactivity phenotype. Final exposure classifications were based on a majority vote across all RBD variants. Final classifications for samples were: 96% negative (277/289), 3.8% cross-reactive (11/289), < 1% likely infected (1/289). N: nucleocapsid, RBD: receptor binding domain, MFI: mean fluorescence intensity. (PDF) [file pone.0353284.s026.pdf]

Healthy Participants

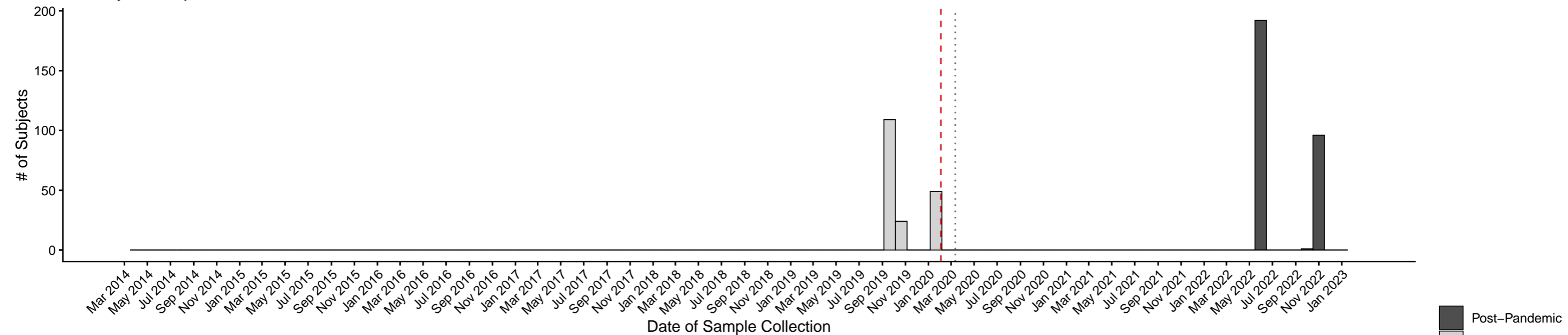

Participants with Cancer

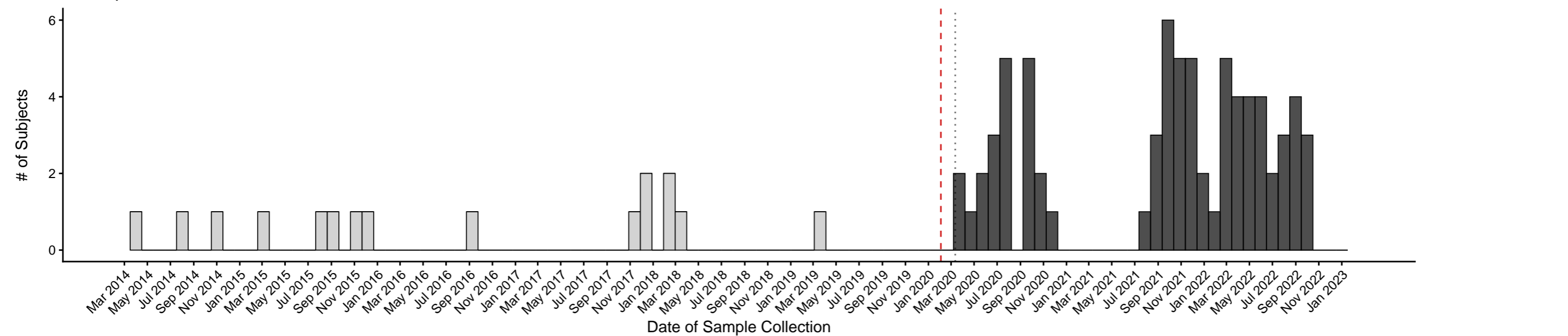

Supplement: S16 Fig — Histograms show dates of plasma sample collection for healthy participants (top row) and participants with cancer (bottom row). The red dashed line indicates the cutoff used to define pre- versus post-pandemic periods (February 1, 2020) and the dotted black line represents the first confirmed case of COVID-19 in Kenya (March 12, 2020). (PDF) [file pone.0353284.s028.pdf]

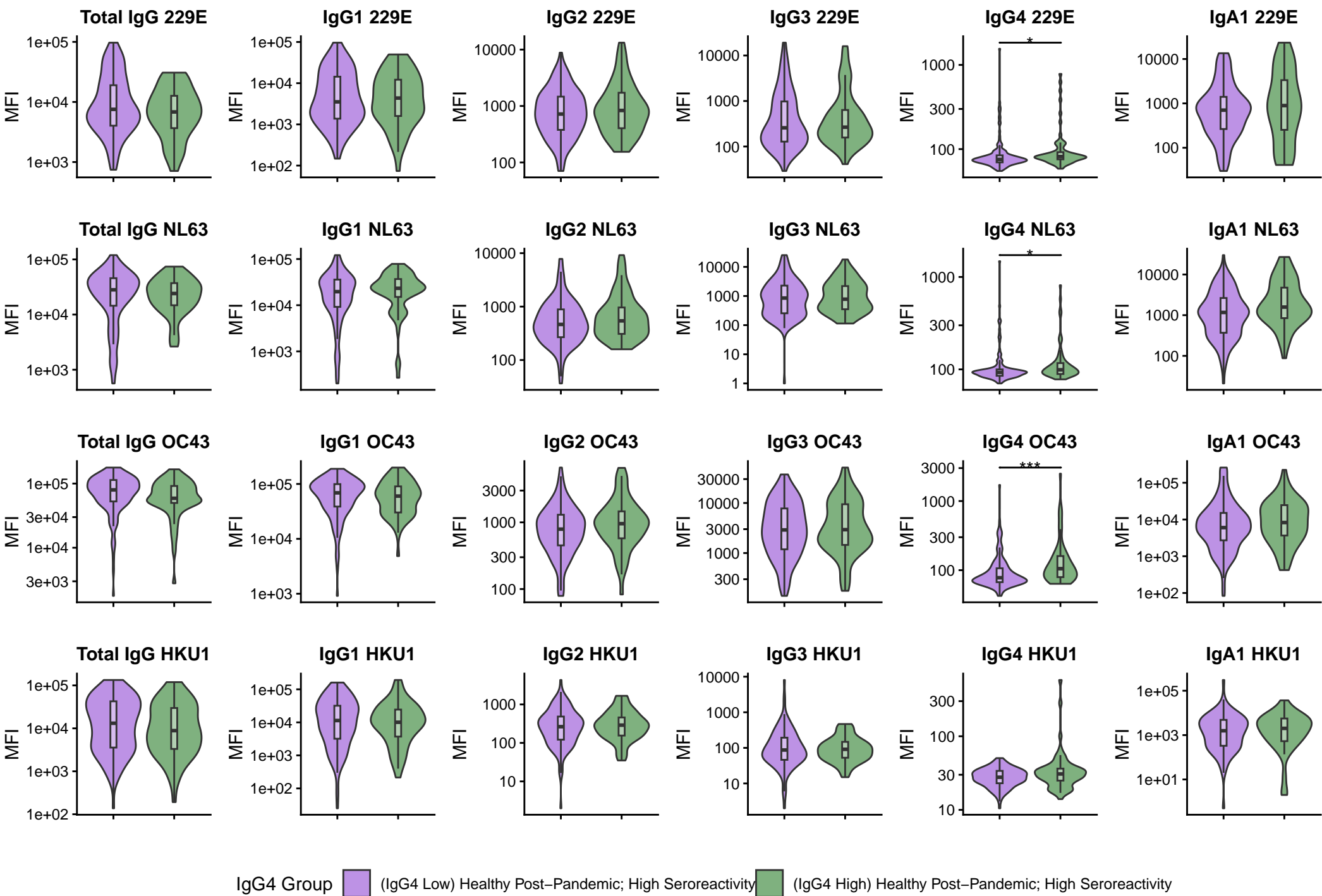

Supplement: S18 Fig — HCoV-specific antibody levels in healthy children sampled post-pandemic with relative higher (green; n = 62) or lower (purple; n = 138) SARS-CoV-2-specific IgG4 levels. Statistical significance was assessed using the Mann-Whitney U test, with multiple comparisons adjusted by the Benjamini-Hochberg procedure (*p < 0.05, **p < 0.01, ***p < 0.001). Plots include only healthy post-pandemic participants deemed high seroreactive (n = 200). MFI: mean fluorescence intensity. (PDF) [file pone.0353284.s030.pdf]

**a**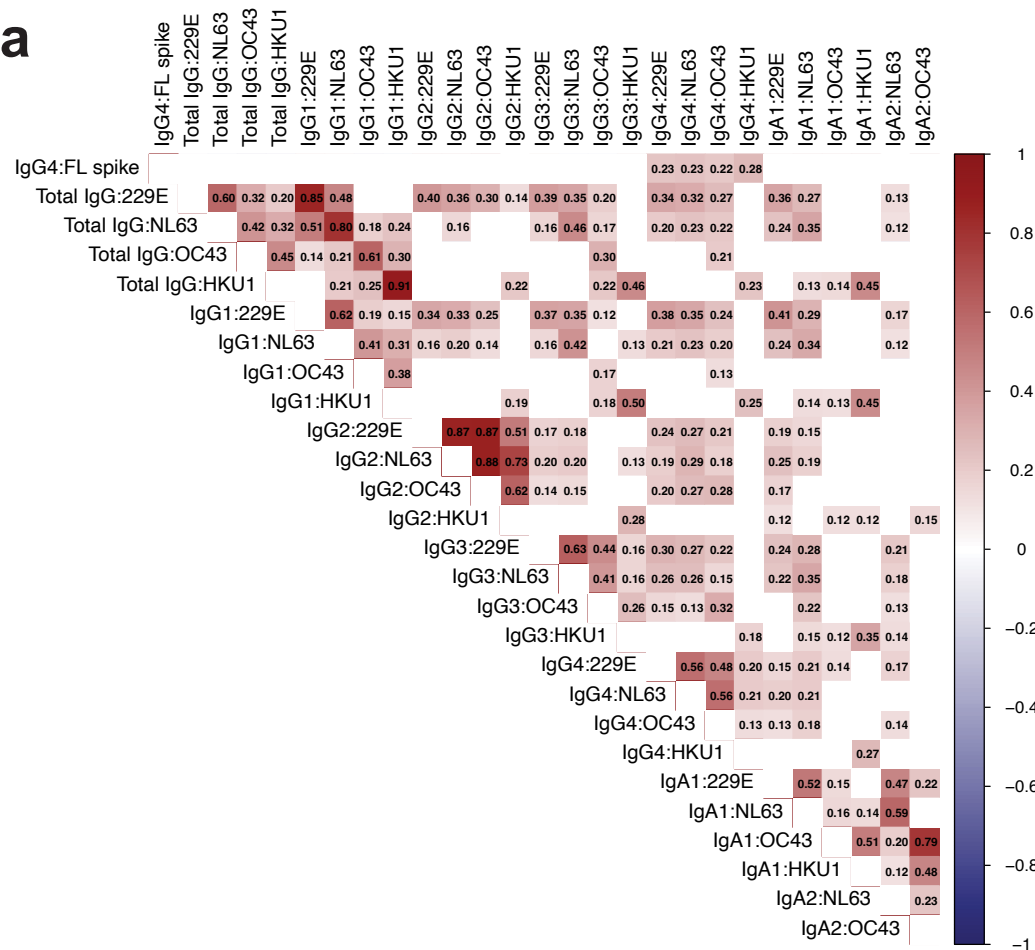**b**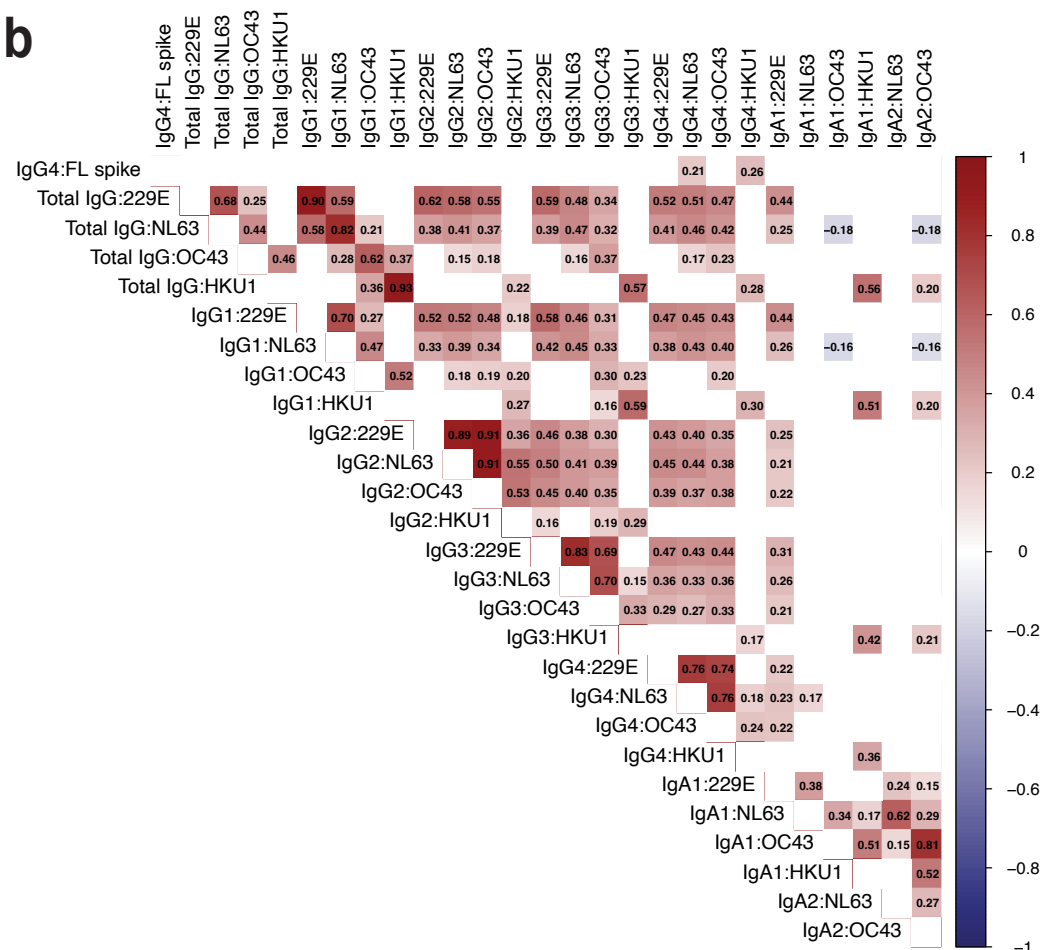

Supplement: S19 Fig — The matrix depicts pairwise Spearman correlations between antibody levels to HCoV and IgG4 response to SARS-CoV-2 spike antigen in healthy post-pandemic (a) and pre-pandemic (b) controls. Only correlations with p < 0.1 after Benjamini-Hochberg adjustment for multiple comparisons are shown. Color and coefficient reflect the direction and magnitude of each pairwise correlation. N: nucleocapsid, RBD: receptor binding domain, FL: full length. (PDF) [file pone.0353284.s031.pdf]

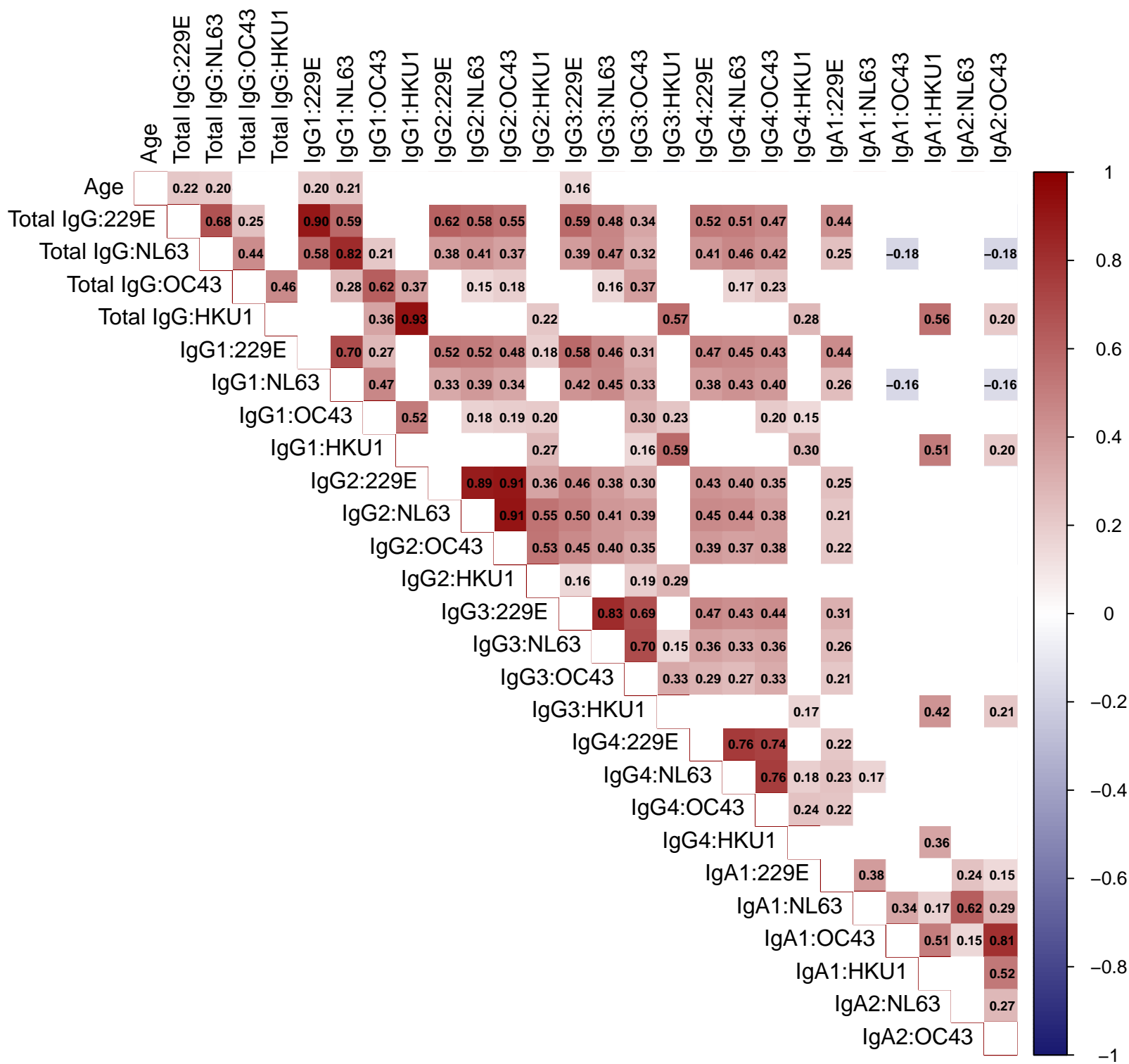

Supplement: S20 Fig — The matrix depicts pairwise Spearman correlations between age and HCoV-specific antibody levels in healthy pre-pandemic samples. Only correlations with p < 0.1 after Benjamini-Hochberg adjustment for multiple comparisons are shown. Color and coefficient reflect the direction and magnitude of each pairwise correlation. (PDF) [file pone.0353284.s032.pdf]

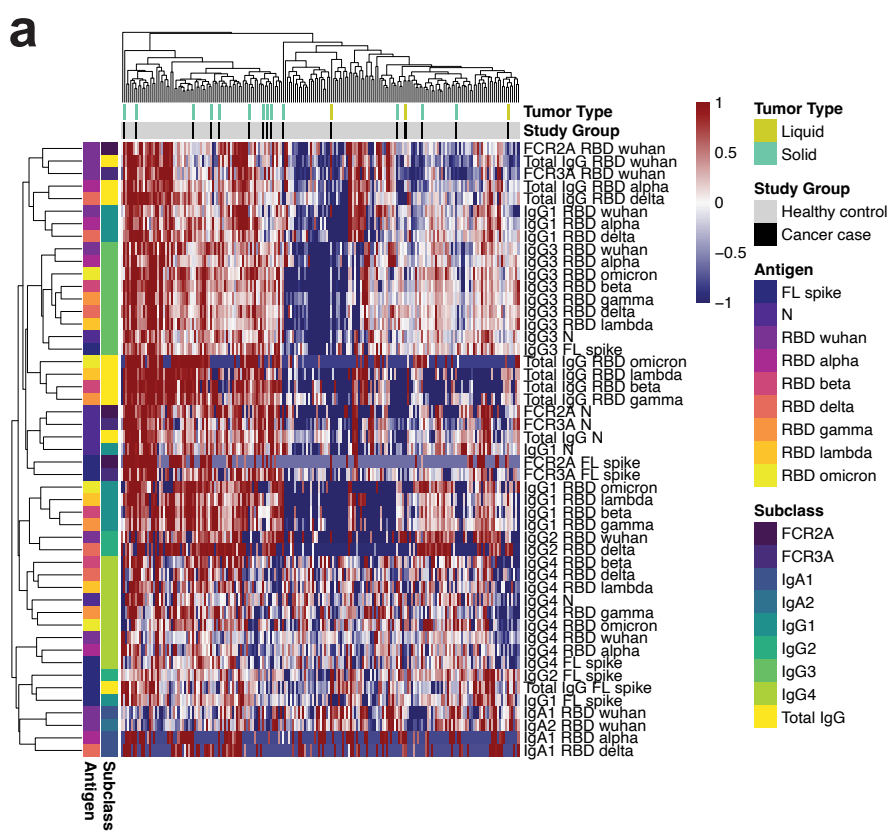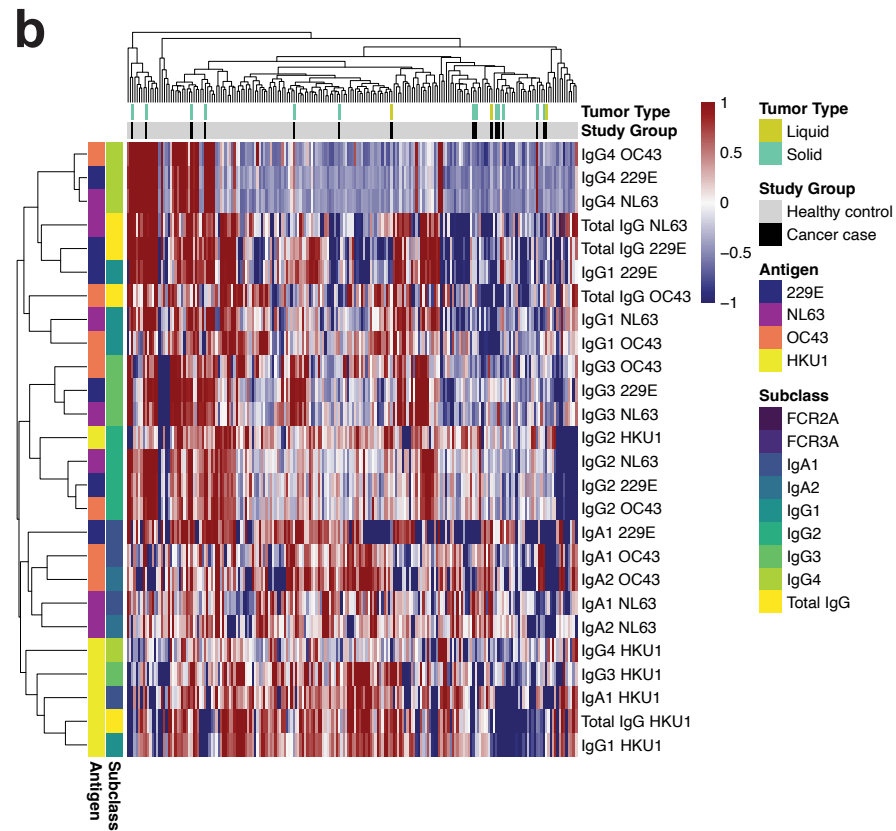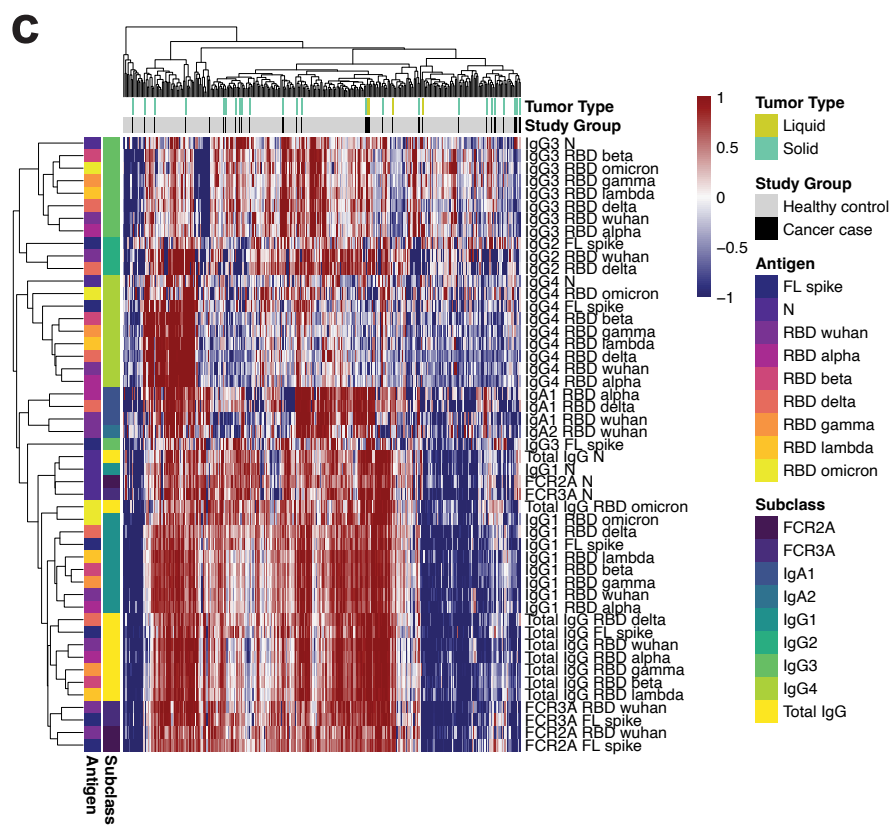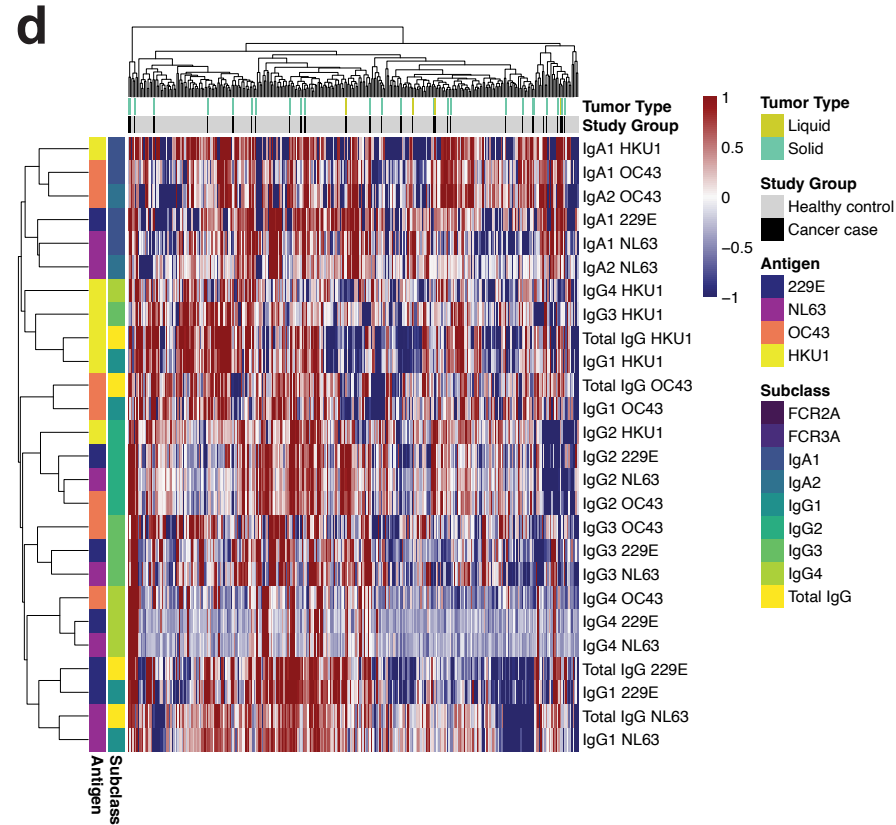

Supplement: S21 Fig — Hierarchical clustering of z-scored SARS-CoV-2 specific (a, c), and HCoV-specific (b,d) antibody features from participants sampled pre-pandemic (top row) or post-pandemic in 2022 (bottom row) with and without cancer. (PDF) [file pone.0353284.s033.pdf]
