# Supplementary material for: Estimating SARS-CoV-2 exposure in asymptomatic hospitalized children with cancer in Western Kenya: A retrospective analysis of serological data
Source: PLoS One. 2026 Jul 10;21(7):e0353284. doi: 10.1371/journal.pone.0353284 (PMC13354098; doi:10.1371/journal.pone.0353284)
Supplement: S2 Fig — Pie charts depict final exposure classifications using seroreactivity thresholds derived from either all pre-pandemic healthy samples (top row) or only those collected before January 1, 2020 (bottom row). Results were highly consistent across definitions, with no evidence of differences in estimated exposure between healthy children and children with cancer sampled in 2022 using Fisher’s exact test (including Jan. 2020 samples: p = 0.83; excluding Jan. 2020 samples: p = 0.73). Estimates in the cancer cohort remained unchanged across thresholds. (PDF) [file pone.0353284.s014.pdf]

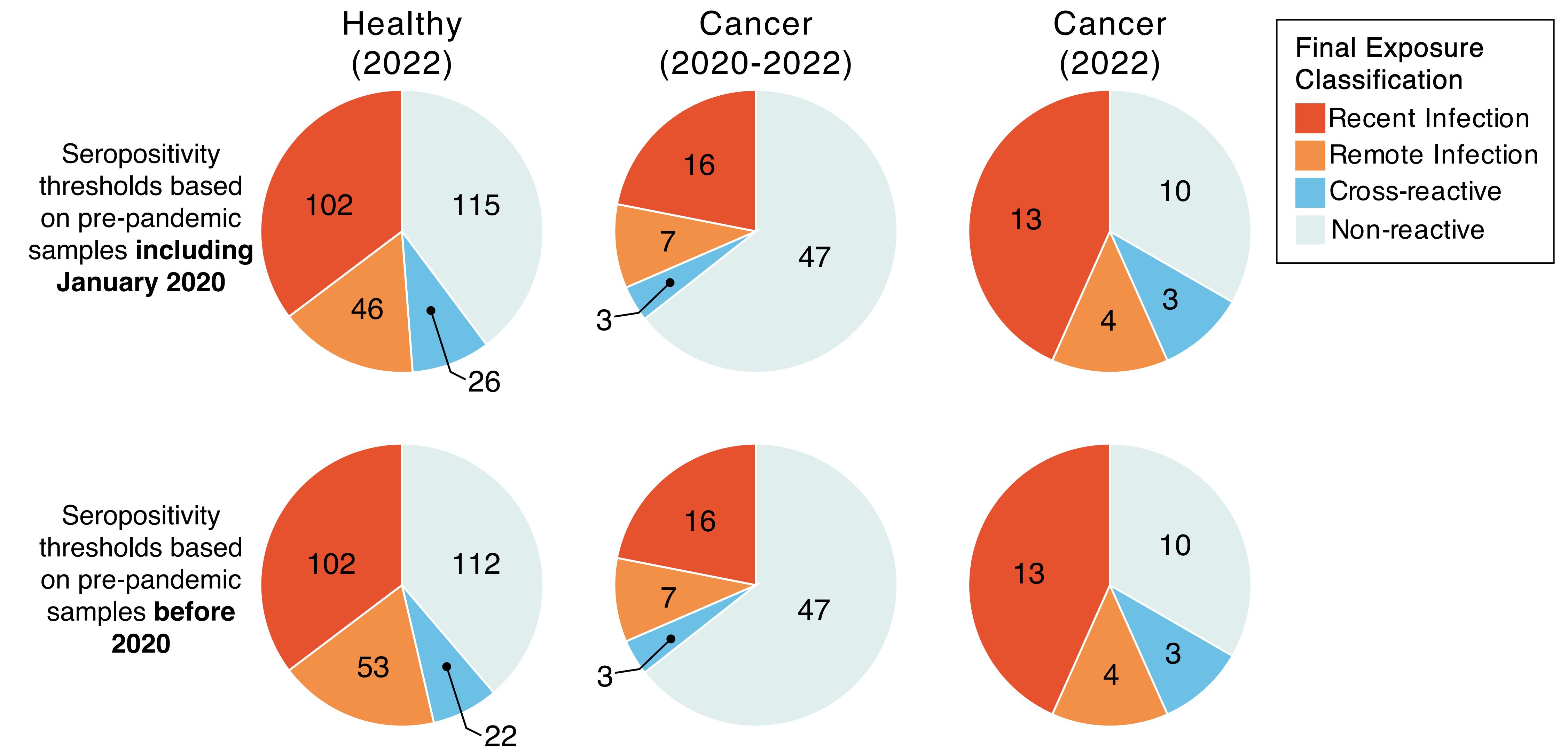

Healthy (2022)
Cancer (2020-2022)
Cancer (2022)
Final Exposure Classification
Recent Infection
Remote Infection
Cross-reactive
Non-reactive
Seropositivity thresholds based on pre-pandemic samples **including January 2020**
102
115
46
26
16
7
3
47
13
10
4
3
Seropositivity thresholds based on pre-pandemic samples **before 2020**
102
112
53
22
16
7
3
47
13
10
4
3
