# Supplementary material for: Estimating SARS-CoV-2 exposure in asymptomatic hospitalized children with cancer in Western Kenya: A retrospective analysis of serological data
Source: PLoS One. 2026 Jul 10;21(7):e0353284. doi: 10.1371/journal.pone.0353284 (PMC13354098; doi:10.1371/journal.pone.0353284)
Supplement: S15 Fig — Pie charts depict exposure estimates for participants when seropositivity is determined using RBD approach as in original analysis (top row) compared to a RBD or N approach (bottom row). P-values from two-proportion z-tests between exposure estimates from healthy children and children with cancer sampled in 2022 were p = 0.71 and p = 0.62 for the top and bottom row, respectively. In the bottom row, participants classified as ‘cross-reactive’ in our original analysis (i.e., those with N-only seropositivity) are now included in the ‘exposed’ group rather than the ‘unexposed’ group. (PDF) [file pone.0353284.s027.pdf]

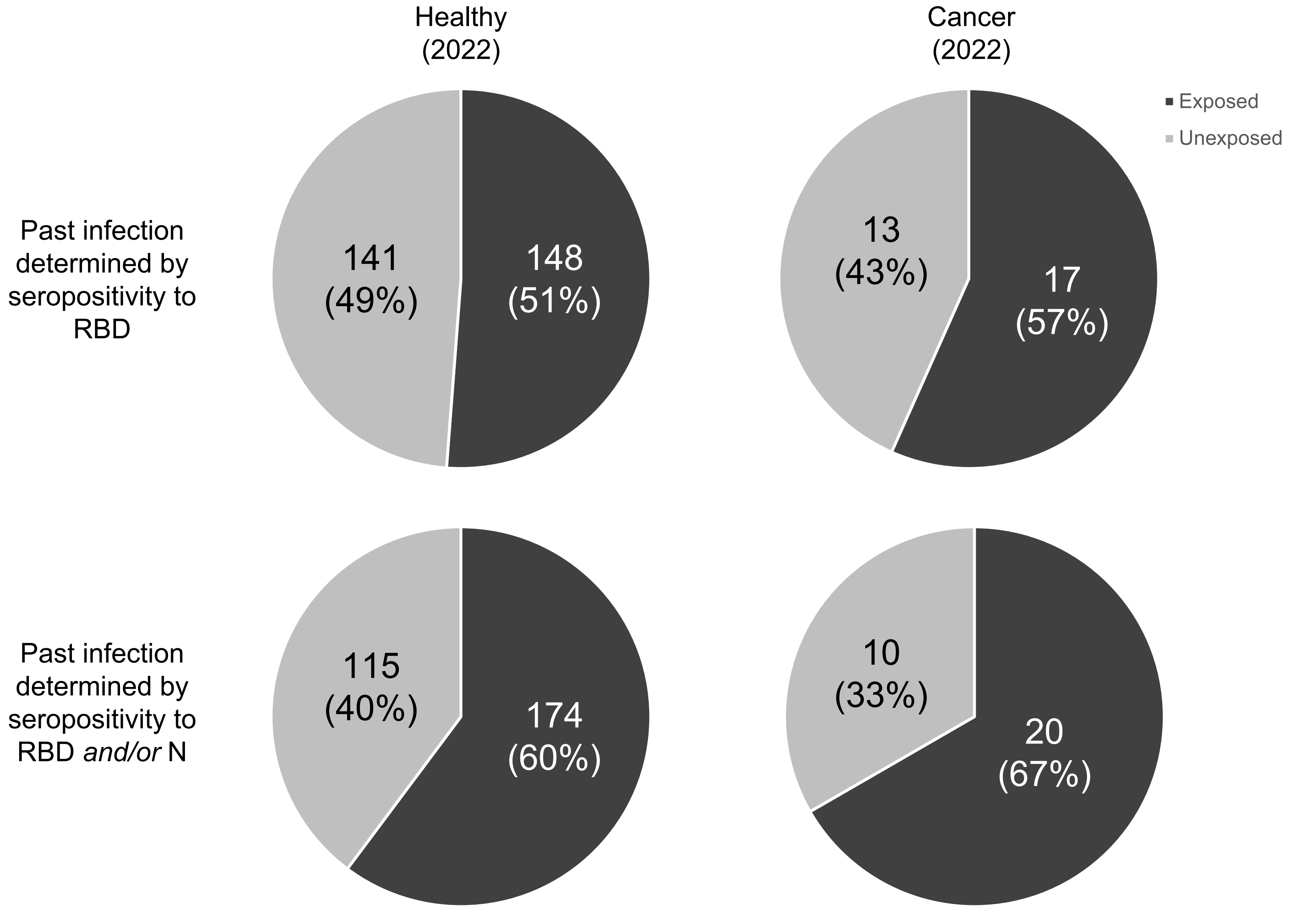
Healthy (2022)
Cancer (2022)
Exposed
Unexposed
Past infection determined by seropositivity to RBD
141 (49%)
148 (51%)
13 (43%)
17 (57%)
Past infection determined by seropositivity to RBD and/or N
115 (40%)
174 (60%)
10 (33%)
20 (67%)
