# Supplementary material for: Estimating SARS-CoV-2 exposure in asymptomatic hospitalized children with cancer in Western Kenya: A retrospective analysis of serological data
Source: PLoS One. 2026 Jul 10;21(7):e0353284. doi: 10.1371/journal.pone.0353284 (PMC13354098; doi:10.1371/journal.pone.0353284)
Supplement: S17 Fig — The heatmaps depicts pairwise correlations between age or sex and SARS-CoV-2-specific antibody levels in pre-pandemic healthy (a), pre-pandemic cancer (b), post-pandemic healthy (c), and post-pandemic cancer (d) participants. Correlations between serology measurements and age or sex were determined by spearman and pearson correlations, respectively. Color and coefficient reflect the direction and magnitude of each pairwise correlation. Significant correlations after Benjamini-Hochberg adjustment for multiple comparisons are denoted by asterisks. (*p < 0.05). N: nucleocapsid, RBD: receptor binding domain, FL: full length. (PDF) [file pone.0353284.s029.pdf]

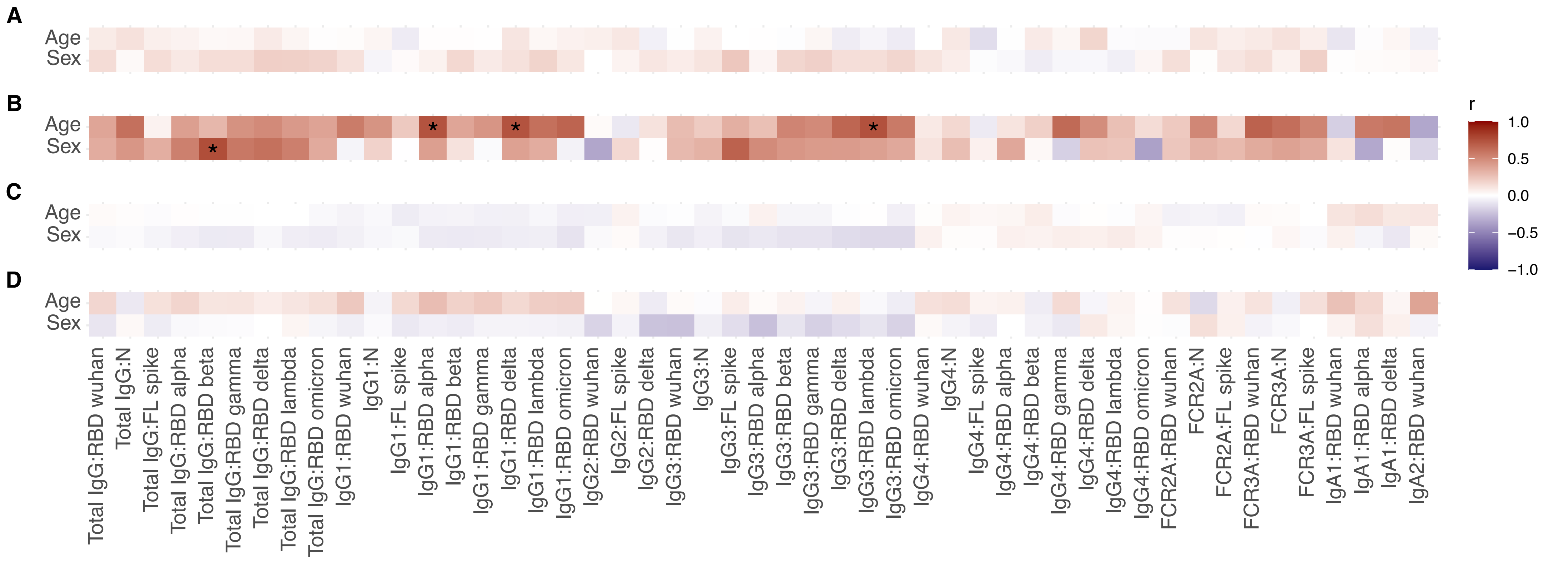
A
B
C
D
Age
Sex
r
1.0
0.5
0.0
−0.5
−1.0
*
Total IgG:RBD wuhan
Total IgG:N
Total IgG:FL spike
Total IgG:RBD alpha
Total IgG:RBD beta
Total IgG:RBD gamma
Total IgG:RBD delta
Total IgG:RBD lambda
Total IgG:RBD omicron
IgG1:RBD wuhan
IgG1:N
IgG1:FL spike
IgG1:RBD alpha
IgG1:RBD beta
IgG1:RBD gamma
IgG1:RBD delta
IgG1:RBD lambda
IgG1:RBD omicron
IgG2:RBD wuhan
IgG2:FL spike
IgG2:RBD delta
IgG3:RBD wuhan
IgG3:N
IgG3:FL spike
IgG3:RBD alpha
IgG3:RBD beta
IgG3:RBD gamma
IgG3:RBD delta
IgG3:RBD lambda
IgG3:RBD omicron
IgG4:RBD wuhan
IgG4:N
IgG4:FL spike
IgG4:RBD alpha
IgG4:RBD beta
IgG4:RBD gamma
IgG4:RBD delta
IgG4:RBD lambda
IgG4:RBD omicron
FCR2A:RBD wuhan
FCR2A:N
FCR2A:FL spike
FCR3A:RBD wuhan
FCR3A:N
FCR3A:FL spike
IgA1:RBD wuhan
IgA1:RBD alpha
IgA1:RBD delta
IgA2:RBD wuhan
