# Supplementary material for: Estimating SARS-CoV-2 exposure in asymptomatic hospitalized children with cancer in Western Kenya: A retrospective analysis of serological data
Source: PLoS One. 2026 Jul 10;21(7):e0353284. doi: 10.1371/journal.pone.0353284 (PMC13354098; doi:10.1371/journal.pone.0353284)
Supplement: S22 Fig — Samples were classified using modified rules requiring at least X of 7 variant-level RBD responses above pre-pandemic thresholds (X = 1-4; X = 4 is equivalent to the original majority voting schema) to classify prior exposure status. For each threshold, samples were assigned to recent, remote, cross-reactive, or non-reactive groups based on N and RBD seropositivity, shown as proportions in pie charts. Exposure assignments did not differ significantly between healthy children and children with cancer sampled in 2022 by Fisher’s exact test across all thresholds (1 variant: p = 0.64; 2 variants: p = 0.15; 3 variants: p = 0.79; 4 variants: p = 0.83). (PDF) [file pone.0353284.s034.pdf]

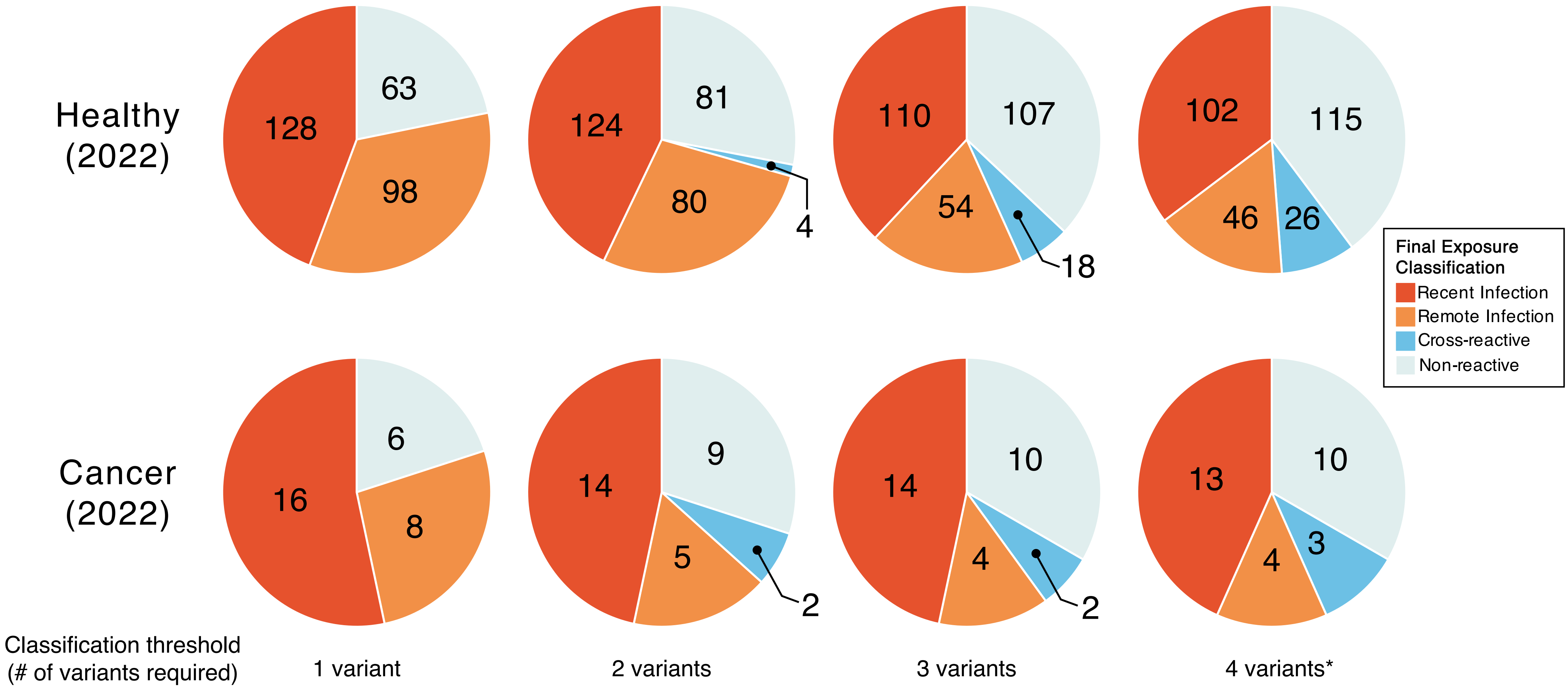

Healthy (2022)
128
63
98
124
81
80
4
110
107
54
18
102
115
46
26
Cancer (2022)
16
6
8
14
9
5
2
14
10
4
2
13
10
4
3
Final Exposure Classification
Recent Infection
Remote Infection
Cross-reactive
Non-reactive
Classification threshold (# of variants required)
1 variant
2 variants
3 variants
4 variants*
